# Developing the Mental Health Ontology: Protocol for a step-wise method to develop an ontology for the mental health domain as part of the GALENOS Project

**DOI:** 10.12688/wellcomeopenres.20701.1

**Published:** 2024-02-15

**Authors:** Paulina M. Schenk, Janna Hastings, Susan Michie

**Affiliations:** 1 Centre for Behaviour Change, University College London, London, England, UK; 2 Institute for Implementation Science in Health Care, University of Zurich, Zürich, Switzerland; 3 School of Medicine, University of St Gallen, St. Gallen, Switzerland

**Keywords:** ontology, framework, classification system, evidence synthesis, living systematic review, GALENOS, mental health, anxiety, depression, psychosis

## Abstract

**Background:**

Research about anxiety, depression and psychosis and their treatments is often reported using inconsistent language, and different aspects of the overall research may be conducted in separate silos. This leads to challenges in evidence synthesis and slows down the development of more effective interventions to prevent and treat these conditions. To address these challenges, the Global Alliance for Living Evidence on aNxiety, depressiOn and pSychosis (GALENOS) Project is conducting a series of living systematic reviews about anxiety, depression and psychosis. An ontology (a classification and specification framework) for the domain of mental health is being created to organise and synthesise evidence within these reviews. It will also be an aid to synthesising evidence in the wider mental health field.

**Aim:**

The aim of the study is to develop a Mental Health Ontology that includes entities with clear and unique labels and definitions to describe and synthesise evidence about mental health.

**Methods:**

We will develop the Mental Health Ontology through six steps: (1) defining the ontology’s scope; (2) identifying, labelling and defining the ontology’s entities for the GALENOS living systematic reviews; (3) identifying and refining entities and their structure by drawing on existing classification frameworks; (4) refining entities via iterative stakeholder consultations regarding the ontology’s clarity and comprehensiveness; (5) formally specifying the relationships between entities in the Mental Health Ontology; and (6) making the ontology machine-readable and available online.

**Conclusion and discussion:**

The Mental Health Ontology supports the formal representation of complex entities within mental health and their relationships. It will enable more explicit and precise communication about mental health across research groups and disciplines, and evidence synthesis across different sources. By being computer readable, the ontology can also be harnessed within algorithms that support automated categorising, retrieving and synthesising evidence.

## Plain language summary

While anxiety, depression and psychosis impact millions of people globally, our current interventions (strategies) to support people with these conditions vary in their effectiveness. We need a shared knowledge base to identify which interventions have worked in the past, and how we can develop better interventions moving forward. The Global Alliance for Living Evidence on aNxiety, depressiOn and pSychosis (GALENOS) aims to address these challenges by conducting systematic reviews relating to anxiety, depression and psychosis. To support these systematic reviews, an ontology (classification framework) of mental health will be developed. This ontology will include concepts (formally called entities) to specify aspects of mental health, along with labels and definitions for these concepts and relationships between them. An ontology of mental health can serve as a shared language and framework to communicate and organise evidence about aspects of mental health, such as mental health symptoms ( *e.g.*, insomnia) or different treatments ( *e.g.*, exercise interventions). The ontology will be developed by: (1) identifying concepts that are needed in systematic reviews of the GALENOS Project, (2) identifying and refining concepts based on existing classification frameworks, (3) refining the ontology based on feedback from relevant experts, (4) specifying the relationship between concepts, and (5) making it computer-readable and available online. This ontology could support clearer communication and understanding of evidence about mental health, thereby contributing to building a shared knowledge base about mental health.

## Background

Anxiety, depression and psychosis affect the well-being of millions of people across the world ( GBD 2019 Mental Disorders Collaborators, 2022). However, current strategies to prevent and treat these conditions vary in their effectiveness ( Leichsenring *et al.*, 2022; Patel *et al.*, 2016). An up-to-date and cumulative knowledge base could support identifying, adapting or developing more effective approaches for prevention and treatment ( Cipriani *et al.*, 2023). To develop such a knowledge base, several challenges in mental health research need to be addressed, including:

1. **The “piece by piece approach” to mental health research** ( Gardner & Kleinman, 2019): Mental health research and treatments often develop in silos, separated by researchers’ education background, disciplines, perspectives and sometimes even ideologies rather than evidence.
2. **Increase in number of publications in mental health** ( Elliott *et al.*, 2021): Efforts to synthesise the literature can quickly become out-of-date, as new evidence is produced at a high speed.
3. **Inconsistent use of language to communicate about specific aspects of mental health** ( *e.g.*, Smoktunowicz *et al.*, 2020): Many constructs in mental health have the same label but different definitions or vice versa have the same definition but different labels, creating challenges for communicating about these constructs and synthesising evidence across different studies.
4. **Lack of focus on studying the mechanisms of mental health interventions** ( Domhardt *et al.*, 2021; Insel & Gogtay, 2014): Studying the causes of mental health issues and the mechanisms through which interventions work can provide evidence for biomarkers for pharmacological, psychological and social interventions and more broadly “why” the interventions work, thereby supporting the translatability of findings across different populations and settings. However, interventions are often evaluated solely in terms of their influence on outcomes rather than their mechanisms.
5. **Lack of focus on studying mental health outcomes that are important to those most affected** ( Nature Editorial, 2018; White *et al.*, 2023): People with lived experience of mental health issues are often not consulted when designing research and studying these issues, leading to their needs being insufficiently addressed in research projects and their outputs.

The Global Alliance for Living Evidence on aNxiety, depressiOn and pSychosis (GALENOS) aims to address these challenges by synthesising and maintaining up-to-date knowledge relating to anxiety, depression and psychosis through a range of living systematic reviews ( Cipriani *et al.*, 2023). The project will include a focus on mental health interventions’ mechanisms and involve experiential advisors at all stages of the project.

To support these systematic reviews, an ****ontology**** for the domain of mental health will be developed (see glossary of bold, italicised terms in Table 1). An ontology is a classification system including representations of ****entities**** (anything that exists in the universe, such as objects and processes) with clear labels and definitions, interconnected by ****relationships**** ( Arp *et al.*, 2015).

Mental health has been defined as “ *a state of mental well-being that enables people to cope with the stresses of life, realize their abilities, learn well and work well, and contribute to their community*” ( WHO, 2022). By defining and categorising a broad range of aspects of mental health and the experiences associated with the conditions in which mental health is impacted, the ontology can encompass broad psychological and experiential views of mental health ( *e.g.*, Johnstone & Boyle, 2018), as well as more traditional diagnostic models of mental health conditions ( Larsen & Hastings, 2018). There are ongoing debates about the best way of classifying mental health conditions, given that people with the same diagnoses can experience very different symptoms, while people with different diagnoses can experience the same or very similar symptoms ( Clark *et al.*, 2017; Conway *et al.*, 2021; Feczko *et al.*, 2019; Robinaugh *et al.*, 2020). Given these debates, the ontology aims to provide a strategy for representing experienced symptoms as entities in addition to representing diagnoses and the potential interrelationships between these. Moreover, different diagnostic systems such as DSM-5, ICD and RDoC ( Clark *et al.*, 2017) will be explicitly supported through cross-references. A key advantage of ontologies is that they can be continually updated based on evidence and feedback ( Arp *et al.*, 2015; He *et al.*, 2018), allowing entities and their relationships to evolve in response to broadening consensus within the mental health field.

The Mental Health Ontology will:

- provide a shared framework to communicate, organise and analyse evidence about aspects of mental health across research teams in GALENOS and beyond;
- be used to strengthen the systematic reviews by helping identify additional search terms and entities to include in extraction sheets;
- allow mapping of the results of human and non-human studies onto the ontology’s entities to synthesise evidence from different sources, thereby increasing the strength of evidence relevant to the discovery of personalised and effective new treatments;
- support the research data portal by informing how research is browsed, categorised, indexed and summarised;
- facilitate the use of reliable machine learning as a step towards enabling more efficient processes to categorise, retrieve and synthesise evidence as it is published in the future. The synthesis methods will necessarily differ at times from those conventionally used in other systematic reviews ( *e.g.*, Cochrane) and will be implemented by acknowledged leaders in their field. As more research enters the system and is classified according to concepts in the ontology, the machine learning will become more attuned to the precise research relevant to each living systematic review. We will use these tools to populate a comprehensive online living evidence summary (see AD-SOLES, for example) ( Hair, 2022).

**Table 1.** Glossary of terms.

Term	Definition	Source
Basic Formal Ontology (BFO)	An upper-level ontology specifying foundational distinctions between different types of entity, such as between continuants and occurrents, developed to support integration, especially of data obtained through scientific research.	Arp *et al.*, (2015)
Entity	Anything that exists, including objects, processes, and their attributes. According to Basic Formal Ontology, entities can be broadly divided into continuants and occurrents. The terms “entity” and “class” can be used interchangeably to refer to the entities represented in an ontology. Classes can be arranged hierarchically by the specification of parent and child classes; see definition of parent class in the glossary	Arp *et al.*, (2015)
Issue tracker	An online log for problems identified by users accessing and using an ontology.	https://docs.github.com/en/issues/tracking-your-work-with-issues/about-issues
Ontology	A standardised representational framework providing a set of entities for the consistent description (or “annotation” or “tagging”) of data and information across disciplinary and research community boundaries.	Arp *et al.*, (2015)
Parent class	An entity within an ontology that is hierarchically related to one or more child classes (subclasses) such that all members of the child class are also members of the parent class, and all properties of the parent class are also properties of the child class.	Arp *et al.*, (2015)
Relationship	The manner in which two entities are connected or linked.	Arp *et al.*, (2015)
ROBOT	An automated command line tool for ontology workflows.	Jackson *et al.* (2019); http://robot.obolibrary.org
Uniform Resource Identifiers (URI)	A string of characters that unambiguously identifies an ontology or an individual entity within an ontology. Having URI identifiers is one of the OBO Foundry principles.	http://www.obofoundry.org/principles/fp-003-uris.html
Versioning	Ontologies that have been released are expected to change over time as they are developed and refined, leading to a series of different files. Consumers of ontologies must be able to specify exactly which ontology files they used to encode their data or build their applications and be able to retrieve unaltered copies of those files in perpetuity. Versioning is one of the OBO Foundry principles.	http://www.obofoundry.org/principles/fp-004-versioning.html
Web Ontology Language (OWL)	A formal language for describing ontologies. It provides methods to model classes of “things”, how they relate to each other and the properties they have. OWL is designed to be interpreted by computer programs and is extensively used in the Semantic Web where rich knowledge about web documents and the relationships between them are represented using OWL syntax.	https://www.w3.org/TR/owl2-quick-reference/

## Methods

### Set up the Mental Health Ontology Advisory Board

***Terms of reference of Advisory Board***

Members of the advisory board will bring their perspectives to the work, recognising that ontologies seek to reflect many perspectives and that consensus is aimed for but not always immediately achieved ( Open Biological and Biomedical Ontology [OBO] Foundry, 2019). They will be invited to attend online meetings to provide feedback during ontology development about the methodology, emerging ontology content and organisation, and ontology-structured evidence. They will also be invited to submit feedback to written documents that will inform ontology development. Based on the number of participants in previous studies to provide feedback on ontologies, we aim to recruit at least 10 members for the advisory board before the initial round of feedback ( Michie *et al.*, 2020; Norris *et al.*, 2020). However, this number is subject to change, with more experts being recruited when people with relevant expertise and lived experience express interest and/or specific expertise are needed.

***Criteria for selection of members***

Selection to reflect representativeness across geography and discipline include:

1. Representation from Global Experiential Advisory Board
2. Volunteers from the Galenos International Advisory Board (including experts with animal and human science content expertise)
3. Individuals who have done work in Mental Health classification or measurement
4. Mental Health organisations to be invited to send a representative
5. Ontology experts

### Ontology development methods

**Figure 1.**
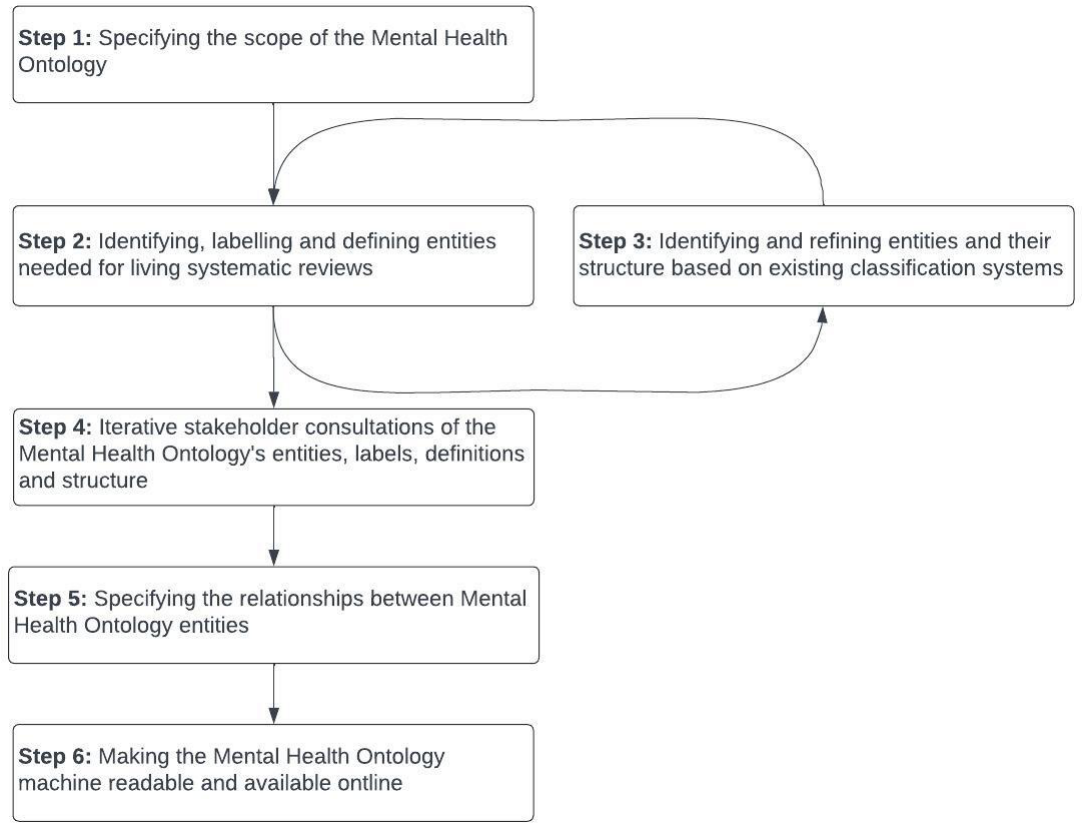
Overview of steps to develop the Mental Health Ontology.

The Mental Health Ontology will be developed in six iterative steps, drawing on the methods applied for the Behaviour Change Intervention Ontology (BCIO; Wright *et al.*, 2020). Figure 1 presents an overview of these steps.

***Step 1: Specifying the scope of the Mental Health Ontology***

The preliminary scope of the Mental Health Ontology will cover: (1) human mental health conceptualisations, including constructs representing symptoms and conditions ( *i.e.*, diagnoses), (2) mental health interventions ( *i.e.*, coordinated sets of activities designed to change specified aspects of mental health) and their delivery, (3) settings in which interventions are delivered, (4) populations to whom interventions are delivered, (5) intervention mechanisms and biomarkers for mental health outcomes, (6) intervention outcomes (including risk prediction) and spillover effects related to mental health and (7) research methods. This scope will be refined during later steps. We will link our human-focused mental health ontology with relevant pre-existing external ontologies that capture and formalise animal research and biomedical research more broadly.

***Step 2: Identifying, labelling and defining entities needed for living systematic reviews***

The GALENOS Project’s ontology development and systematic review teams will work together to identify and refine entities that will be used when searching for studies, extracting data and storing data as part of the living systematic reviews. When the systematic review teams start conducting their reviews, they will share their search terms and categories included in their data extraction sheets with the ontology development team. To capture these terms and categories in the Mental Health Ontology, the ontology development team will first check if a relevant entity is already included in the Mental Health Ontology. If not, the team will identify relevant entities from existing ontologies or develop new entities. Entities from other ontologies will be identified by using specialist ontology databases, *e.g.*, the Ontology Lookup Service ( European Bioinformatics Institute, 2019), and where appropriate, these entities will be reused or cross-referenced in the Mental Health Ontology. New entities will be developed, labelled and defined by drawing on mental health classification systems or dictionaries, and assigned unique alphanumeric identifiers. Synonyms will be added to entities to ensure that variations of a term are fully captured in the Mental Health Ontology. Next, the ontology development team will share relevant entities, their labels, definitions and synonyms with the systematic review teams for feedback on whether each entity appropriately captures the term or category of interest. Where teams suggest changes to entities, these entities will be updated in the ontology. If the entities have previously been applied by other systematic review teams, they will be informed of the changes and given the opportunity to raise issues with these changes. To avoid inter-dependencies that will delay the systematic reviews, the systematic review teams can use the terms or categories they developed for data extraction, once these are checked with the ontology developers. The ontology developers can map these terms or categories and the relevant data onto the ontology entities after the reviews have started.

*Capturing entities beyond mental health*

For terms or categories needed for systematic reviews that are beyond the Mental Health Ontology’s scope ( *e.g.*, terms or categories not specific to humans and related to animals more broadly, or drugs and biochemistry), we will link our ontology to relevant entities in external ontologies. For instance, we will search for animal-related entities in external ontologies and share these with relevant systematic review teams for feedback on whether these capture terms and categories of interest. We will work closely with the developers of these external ontologies in order to jointly determine the appropriate way to link their ontologies with ours ( *e.g.*, bridging relationships) to capture the relevant aspects for a given study. We will request additional content when it is missing in the external ontologies. The relevant external content will be available for the GALENOS database annotations.

***Step 3: Identifying and refining entities and their structure based on existing classification systems***

Parallel to developing entities used to map the living systematic review, we will identify, refine, and structure entities based on existing classification systems. This will include surveying existing classification systems on mental health by: (1) asking the Mental Health Ontology Advisory Board to suggest relevant classification systems, (2) searching for such classification systems on domain databases and associated controlled vocabularies, *e.g.*, MeSH, and (3) searching relevant ontologies on specialist ontology databases, e.g., the Ontology Lookup Service ( European Bioinformatics Institute, 2019). We will share the final list of identified classification systems with the Mental Health Ontology Advisory Board to allow them to make additions.

From these classification systems, we will extract constructs and their definitions and group any constructs with overlapping definitions together to create unique entities for the ontology or reuse relevant entities from other ontologies. When constructs are already captured by entities included in the Mental Health Ontology in Step 2, the ontology team will review entity definitions to ensure that they sufficiently capture constructs from existing classification system. Changes to entities will be communicated with relevant systematic review teams. For the structure of the upper-level entities in the Mental Health Ontology, we will reuse or adapt relevant entities from the BCIO, which drew extensively on stakeholder feedback ( Wright *et al.*, 2020).

***Step 4: Iterative stakeholder consultations of the Mental Health Ontology***

Stakeholder consultations of the Mental Health Ontology will be conducted to ensure that the ontology: (1) clearly reflects entities important to people experiencing related mental health issues ( *i.e.*, people with lived experience), (2) captures a broader scientific consensus in the mental health field, and (3) meets the needs of potential ontology users ( OBO Foundry, 2019; Wright *et al.*, 2020). Participants will be recruited by (1) inviting members of the Mental Health Ontology Advisory Board and (2) asking these members to suggest individuals or groups with relevant expertise. As the ontology will be extensively updated throughout the project’s lifecycle, stakeholders will be consulted at multiple stages of development, approximately every 9–12 months. The consultations will be carried out with two groups of participants who can provided different types of feedback on the ontology: (1) domain experts ( *e.g.*, researchers working in the mental health domain) and (2) experiential advisors.

*Stakeholder consultation with domain experts*

We aim to recruit at least 10 participants with broad theoretical knowledge and expertise relating the mental health field, and at least three participants with expertise relating to ontologies. The number of participants is considered appropriate based on the development of ontologies part of the BCIO, which included 3–29 participants in their stakeholder consultations ( Michie *et al.*, 2020; Norris *et al.*, 2020).

Participants will be provided with general training that covers: (1) what an ontology is and (2) an overview of the Mental Health Ontology. They will then be presented parts of the Mental Health Ontology and invited to participate in a survey to provide feedback on the ontology. Feedback will be prompted about:

1). The clarity of the ontology: Whether any entity, label or definition is unclear
2). The comprehensiveness of the ontology as a whole or the specific part for which review feedback is being sought: Whether any entities are missing from the ontology

Where participants indicate that parts of the ontology are unclear or entities are missing, they will be asked to suggest relevant changes to improve the ontology. Participants will also be asked if they have any additional feedback which was not prompted by other survey questions.

Each piece of feedback from the participants will be recorded and reviewed by a researcher (PS) to propose changes to the ontology. The relevant feedback and proposed changes will be discussed among the three researchers leading the Mental Health Ontology’s development (JH, SM & PS). We will record decisions regarding how each piece of feedback will be addressed.

*Stakeholder consultations with participants with lived experience*

To reflect the knowledge of people with lived experience of mental health issues in the ontology ( National Institute for Health Research [NIHR], 2019) we will carry out iterative rounds of feedback involving people with lived experience. The process to recruit participants and the materials for collecting feedback from people with lived experience will be co-developed with at least one experiential advisor member of the Mental Health Ontology Advisory Board. In addition to the entity labels and definitions, more user-friendly parts of the ontology ( *e.g.*, informal definitions, which are simpler than formal ontology definitions and in Plain English language where possible) will be shared with the participants. Consistent with the stakeholder consultations with domain experts, we will prompt feedback about the clarity and comprehensiveness of the ontology. In these consultations, when participants indicate that parts of the ontology need changing, they will also be asked to make suggestions to improve the ontology.

Each piece of feedback from participants will be treated as described for the stakeholder consultations with domain experts but with additional steps to better align the principles of co-production ( NIHR, 2019). Once the ontology developers have agreed on changes to the ontology, the proposed changes will be shared with consultation participants. They will be asked whether the changes are appropriate or additional changes are needed to capture their knowledge and experience of relevant mental health issues. This second round of feedback will then be discussed by the ontology developers, and changes will be made accordingly.

***Step 5: Specifying the relationships between Mental Health Ontology entities***

The ontology development team will discuss, specify and refine the relationships between entities in the Mental Health Ontology. Common relationships ( *e.g.*, “is_a” and “has_part”) will be used from the widely used upper-level ontologies ****Basic Formal Ontology**** and the Relation Ontology ( Smith *et al.*, 2005).****** To structure the ontology, each entity will be linked to a ****parent class**** with a hierarchical “is_a” relationship ( Arp *et al.*, 2015; Smith *et al.*, 2005). For instance, the entity “motivation” will have an “is_a” relationship to its parent class “mental process”: motivation “is_a” mental process. The team will also discuss whether any new relations need to be specified between entities to structure the ontology and, if so, develop such relations.

***Step 6: Making the Mental Health Ontology machine-readable and available online***

We will develop the Mental Health Ontology as a spreadsheet of entities: Each entity will be organised as a separate row with a primary label and definition, unique alphanumeric identifier ( *i.e.*, ****Uniform Resource Identifier [URI]****; e.g., BCIO:01023), relationships, and if available, synonyms, informal definitions and examples. These inputs ( *e.g.*, label and definition) will be organised into separate columns. When the ontology’s content is ready for its initial release, we will convert this content to ****Web Ontology Language (OWL)**** ( Antoniou & van Harmelen, 2004) format. In this standard format, the ontology can be viewed and visualised within ontology software, such as Protégé ( Musen, 2015), and becomes compatible with other ontologies. For the conversion to OWL, we will use the ****ROBOT**** ontology toolkit library ( Jackson *et al.*, 2019), which supports creating well-formatted ontologies from spreadsheet-format templates. The ROBOT template is a comma-separated values (CSV) file that is prepared from the primary ontology spreadsheets by adding instructions to the template header about how spreadsheet columns are to be converted into OWL and metadata attributes. The Mental Health Ontology’s OWL version will be stored on the ***GitHub*** repository of the project, as this repository supports versioning of the ontology, *i.e.*, it keeps a record of different versions of the ontology and any updates made. GitHub also has an ****issue tracker**** that enables ontology users to submit any issues with the ontology and ontology developers to respond to these issues.

### Applying the ontology to develop tools for data searching, visualising, extraction and synthesis, and partial automation of these processes

The Mental Health Ontology will be used for annotations of the “living evidence” extracted from the literature and stored in the project’s open coded database. The systematic review publications will be linked to this database, and will be regularly updated, allowing new data to be retrieved and displayed ( *e.g.*, as plots) as part of the living systematic review. The ontology will also be used to develop tools and algorithms to support interoperability with other knowledge resources, enhanced searching, browsing and navigating of the evidence database, and ontology-based summarising and visualising the data. In conjunction with language models and the data from living systematic reviews carried out early in the GALENOS Project’s lifecycle, the ontology will also be applied to develop and test structured search strategies for later systematic reviews. Thereby, the ontology development team aims to deliver:

1. A mental health ontology that is interoperable to enable more discoverable and translatable evidence across various sources, including early phase and late phase trials
2. Ontology-based algorithms to enable evidence searching, visualisation and querying
3. An open, coded and queryable database of relevant studies, characteristics of studies, risk of bias assessments and results data, richly linked to ontologies for interoperability

## Ethics

Ethical approval was granted by University College London’s ethics committee (CEHP/2020/579) in 2020. Participant informed written consent will be sought at the beginning of each stakeholder consultation.

## Study status

We have specified the initial scope of the ontology (Step 1), started inviting participants to join the Mental Health Advisory Board (Step 2), and drafted the initial entities for the first three systematic reviews as part of the GALENOS Project (Step 3).

## Conclusion

The Mental Health Ontology will be developed to serve as a shared framework to categorise, label and define entities relating to mental health research within the GALENOS Project and beyond. The entities will include key constructs for diagnoses of conditions affecting mental health, experiences related to mental health, mental health interventions, their target populations and settings, intervention mechanisms and biomarkers for mental health outcomes, intervention outcomes and research methods. As these groups of constructs will each be elaborated and categorised in the ontology, it will enable the representation of entities relevant to different perspectives in mental health research and the integration of evidence from sources informed by such perspectives.

This ontology will be used to support the GALENOS living systematic reviews’ searches and data extraction, synthesis, analysis and visualisation. We will develop this ontology iteratively, updating it based on the needs of living systematic reviews and stakeholder feedback. When living systematic reviews include evidence about entities beyond the scope of the Mental Health Ontology, the ontology-based database will draw on relevant entities in external ontologies, *e.g.*, about animal research. As ontologies are computer readable, some of these processes can also be partially automated in the project lifecycle or refined to be fully automated after the project.

## Data Availability

No data are associated with this article.
