# Towards an ontology of mental health: Protocol for developing an ontology to structure and integrate evidence regarding anxiety, depression and psychosis

**DOI:** 10.12688/wellcomeopenres.20701.2

**Published:** 2024-11-14

**Authors:** Paulina M. Schenk, Janna Hastings, Micaela Santilli, Jennifer Potts, Jaycee Kennett, Claire Friedrich, Susan Michie

**Affiliations:** 1 Centre for Behaviour Change, University College London, London, England, UK; 2 Institute for Implementation Science in Health Care, University of Zurich, Zürich, Switzerland; 3 School of Medicine, University of St Gallen, St. Gallen, Switzerland; 4 Department of Psychiatry, University of Oxford, Oxford, UK; 5 Oxford Precision Psychiatry Lab, NIHR Oxford Health Biomedical Research Centre, Oxford, UK

**Keywords:** ontology, framework, classification system, evidence synthesis, living systematic review, GALENOS, mental health, anxiety, depression, psychosis

## Abstract

**Background:**

Research about anxiety, depression and psychosis and their treatments is often reported using inconsistent language, and different aspects of the overall research may be conducted in separate silos. This leads to challenges in evidence synthesis and slows down the development of more effective interventions to prevent and treat these conditions. To address these challenges, the Global Alliance for Living Evidence on aNxiety, depressiOn and pSychosis (GALENOS) Project is conducting a series of living systematic reviews about anxiety, depression and psychosis. An ontology (a classification and specification framework) for the domain of mental health is being created to organise and synthesise evidence within these reviews and present them in a structured online data repository.

**Aim:**

This study aims to develop an ontology of mental health that includes entities with clear labels and definitions to describe and synthesise evidence about mental health, focusing on anxiety, depression and psychosis.

**Methods:**

We will develop and apply the GALENOS Mental Health Ontology through eight steps: (1) defining the ontology’s scope; (2) identifying, labelling and defining the ontology’s entities for the GALENOS living systematic reviews; (3) structuring the ontology’s upper level (4) refining entities via iterative stakeholder consultations regarding the ontology’s clarity and scope; (5) formally specifying the relationships between entities in the Mental Health Ontology; (6) making the ontology machine-readable and available online; (7) integrating the ontology into the data repository; and (8) exploring the ontology-structured repository’s usability.

**Conclusion and discussion:**

The Mental Health Ontology supports the formal representation of complex upper-level entities within mental health and their relationships. It will enable more explicit and precise communication and evidence synthesis about anxiety, depression and psychosis across the GALENOS Project’s living systematic reviews. By being computer readable, the ontology can also be harnessed within algorithms that support automated categorising, linking, retrieving and synthesising evidence.

## Plain language summary

While anxiety, depression and psychosis impact millions of people globally, our current interventions (strategies) to support people with these conditions vary in their effectiveness. We need a shared knowledge base to identify which interventions have worked in the past, and how we can develop better interventions moving forward. The Global Alliance for Living Evidence on aNxiety, depressiOn and pSychosis (GALENOS) aims to address these challenges by conducting systematic reviews relating to anxiety, depression and psychosis. To support these systematic reviews, an ontology (classification framework) of mental health will be developed. This ontology will include concepts (formally called entities) to specify aspects of mental health, along with labels and definitions for these concepts and relationships between them. An ontology of mental health can serve as a shared language and framework to communicate and organise evidence about aspects of mental health, such as mental health symptoms ( *e.g.*, insomnia) or different treatments ( *e.g.*, exercise interventions). The ontology will be developed by: (1) identifying concepts that are needed in systematic reviews of the GALENOS Project, (2) identifying and refining concepts based on existing classification frameworks, (3) refining the ontology based on feedback from relevant experts, (4) specifying the relationship between concepts, and (5) making it computer-readable and available online. This ontology could support clearer communication and understanding of evidence about mental health, thereby contributing to building a shared knowledge base about mental health.

## Background

Mental health conditions (e.g., anxiety, depression and psychosis) affect the well-being of millions of people across the world ( GBD 2019 Mental Disorders Collaborators, 2022). However, current strategies to prevent and treat these conditions vary in their effectiveness ( Leichsenring *et al.*, 2022; Patel *et al.*, 2016). An up-to-date and cumulative knowledge base could support identifying, adapting or developing more effective approaches for prevention and treatment ( Cipriani *et al.*, 2023). To develop such a knowledge base, several challenges in mental health research need to be addressed, including:

1. **The “piece by piece approach” to mental health research** ( Gardner & Kleinman, 2019): Mental health research and treatments often develop in silos, separated by researchers’ education background, disciplines, perspectives and sometimes even ideologies rather than evidence.
2. **Increase in number of publications in mental health** ( Elliott *et al.*, 2021): Efforts to synthesise the literature can quickly become out-of-date, as new evidence is produced at a high speed.
3. **Inconsistent use of language to communicate about specific aspects of mental health** ( *e.g.*, Smoktunowicz *et al.*, 2020): Many constructs in mental health have the same label but different definitions, or vice versa, have the same definition but different labels, creating challenges for communicating about these constructs and synthesising evidence across different studies.
4. **Lack of focus on studying the mechanisms of mental health interventions** ( Domhardt *et al.*, 2021; Insel & Gogtay, 2014): Studying the causes of mental health issues and the mechanisms through which interventions work can provide evidence for biomarkers for pharmacological, and targets for psychological and social interventions and more broadly ‘why’ the interventions work, thereby supporting the translatability of findings across different populations and settings. However, interventions are often evaluated solely in terms of their influence on outcomes rather than their mechanisms.
5. **Lack of focus on studying mental health outcomes that are important to those most affected** ( Nature Editorial, 2018; White *et al.*, 2023): People with lived experience of mental health issues are often not consulted when designing research and studying these issues, leading to their needs being insufficiently addressed in research projects and their outputs.

The Global Alliance for Living Evidence on aNxiety, depressiOn and pSychosis (GALENOS) aims to address these challenges by synthesising and maintaining up-to-date knowledge relating to anxiety, depression and psychosis through a range of living systematic reviews ( Cipriani *et al.*, 2023). GALENOS is a global project, funded by the Wellcome Trust, that aims to identify promising routes of new treatments, novel diagnostic tools and more accurate predictions within anxiety, depression and psychosis, by evaluating existing evidence across animal and human data. The three broad mental health conditions (anxiety, depression and psychosis) have been prioritised by the Wellcome Trust, as they are among the top contributors to the global burden of disease ( GBD 2019 Mental Disorders Collaborators, 2022; https://wellcome.org/what-we-do/mental-health).

This project has global reach, with a Leadership Team of members from Australia, Europe, Japan and South Africa, and Global Lived Experience Advisory Board with members from Canada, India, Nigeria, Philippines and Zimbabwe. They include clinicians, experts by lived experience, and researchers, with expertise across data science, psychology and psychiatry. Detailed information about the GALENOS Project can be found on the project website ( https://www.galenos.org.uk/about), as well as in the protocol for the overarching project ( https://mentalhealth.bmj.com/content/ebmental/26/1/e300759.full.pdf).

**Figure 1.**
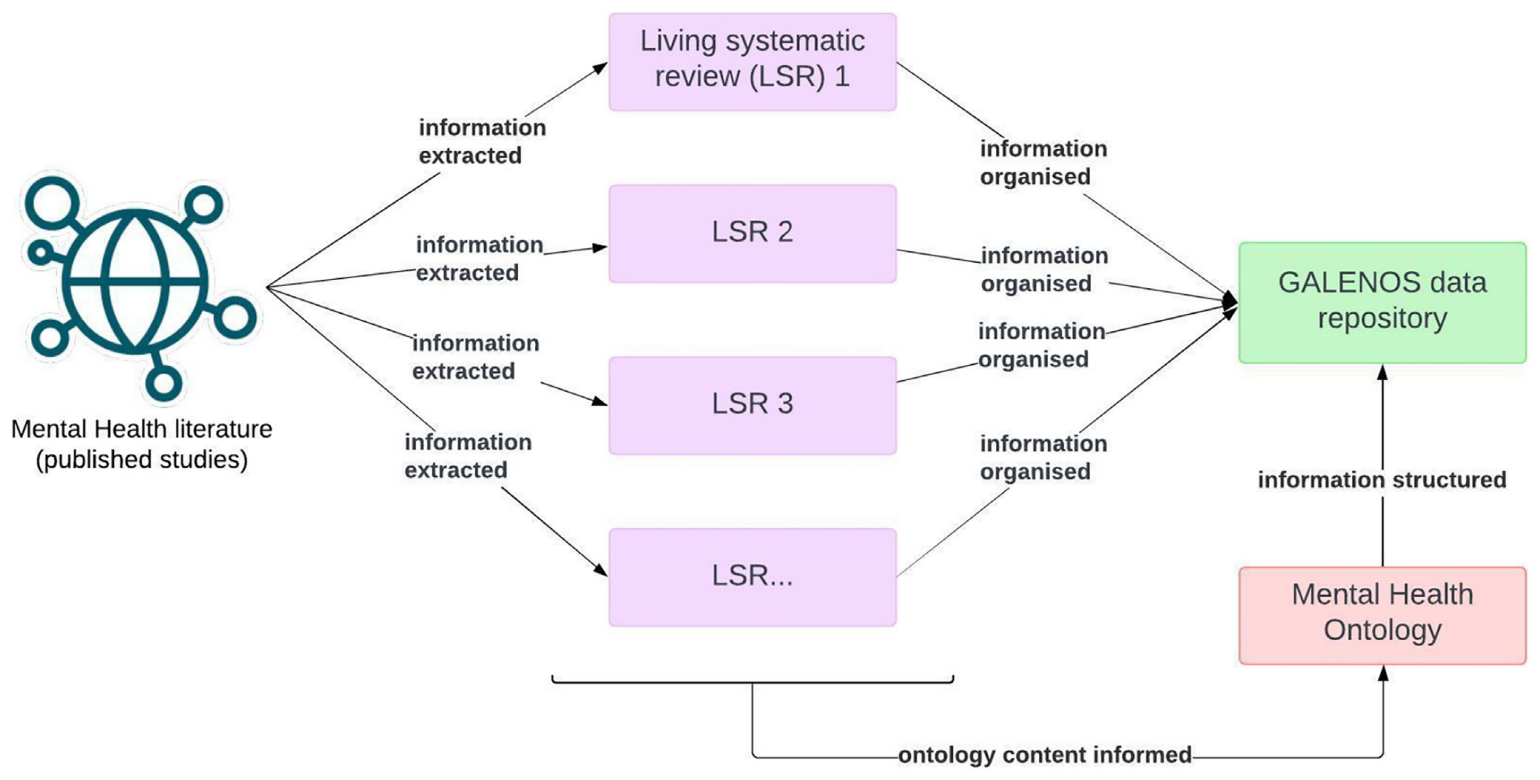
Overview of the GALENOS Project’s workflow.

A key outcome of the project will be an online repository to present and maintain the data and findings of all living systematic reviews and each of their updates, allowing people to review and reuse this data in the future. To help structure the GALENOS Project’s repository, we will develop an ***ontology*** for the domain of mental health, with specific focus on anxiety, depression and psychosis (see glossary of bold, italicised terms in Table 1). An ontology is a classification system including representations of ***entities*** (anything that exists in the universe, such as objects and processes) with clear labels and definitions, interconnected by ***relationships*** ( Arp *et al.*, 2015). Ontologies are being increasingly recognised as tools that can facilitate a shared language to communicate about and help integrate evidence across behavioural and social sciences ( National Academies of Sciences, 2022; Sharp *et al*., 2023). In the GALENOS Project, the ontological entities are developed or reused from other ontologies to organise constructs for which data are extracted in the systematic reviews. Constructs that overlap across systematic reviews can be identified and linked. The ontology serves as a framework to consistently label and define constructs, link relevant constructs and synthesise findings across systematic reviews. Figure 1 presents a workflow of the GALENOS Project, indicating where the ontology fits in.

**Table 1.** Glossary of terms.

**Term**	**Definition**	**Source**
Basic Formal Ontology (BFO)	An upper-level ontology specifying foundational distinctions between different types of entity, such as between continuants and occurrents, developed to support integration, especially of data obtained through scientific research.	Arp *et al.*, (2015)
Entity	Anything that exists, including objects, processes, and their attributes. According to Basic Formal Ontology, entities can be broadly divided into continuants and occurrents. The terms ‘entity’ and ‘class’ can be used interchangeably to refer to the entities represented in an ontology. Classes can be arranged hierarchically by the specification of parent and child classes; see definition of parent class in the glossary	Arp *et al.*, (2015)
Issue tracker	An online log for problems identified by users accessing and using an ontology.	https://docs.github.com/en/issues/tracking-your-work- with-issues/about-issues
Ontology	A standardised representational framework providing a set of entities for the consistent description (or ‘annotation’ or ‘tagging’) of data and information across disciplinary and research community boundaries.	Arp *et al.*, (2015)
Parent class	An entity within an ontology that is hierarchically related to one or more child classes (subclasses) such that all members of the child class are also members of the parent class, and all properties of the parent class are also properties of the child class.	Arp *et al.*, (2015)
Relationship	The manner in which two entities are connected or linked.	Arp *et al.*, (2015)
ROBOT	An automated command line tool for ontology workflows.	Jackson *et al.* (2019); http://robot.obolibrary.org
Uniform Resource Identifiers (URI)	A string of characters that unambiguously identifies an ontology or an individual entity within an ontology. Having URI identifiers is one of the OBO Foundry principles.	http://www.obofoundry.org/principles/fp-003-uris.html
Versioning	Ontologies that have been released are expected to change over time as they are developed and refined, leading to a series of different files. Consumers of ontologies must be able to specify exactly which ontology files they used to encode their data or build their applications and be able to retrieve unaltered copies of those files in perpetuity. Versioning is one of the OBO Foundry principles.	http://www.obofoundry.org/principles/fp-004- versioning.html
Web Ontology Language (OWL)	A formal language for describing ontologies. It provides methods to model classes of ‘things’, how they relate to each other and the properties they have. OWL is designed to be interpreted by computer programs and is extensively used in the Semantic Web where rich knowledge about web documents and the relationships between them are represented using OWL syntax.	https://www.w3.org/TR/owl2-quick-reference/

In the context of this work, mental health has been defined as “ *a state of mental well-being that enables people to cope with the stresses of life, realize their abilities, learn well and work well, and contribute to their community*” ( WHO, 2022). By defining and categorising a broad range of aspects of mental health and the experiences associated with the conditions in which mental health (e.g., anxiety, depression and psychosis) is impacted, the ontology can encompass broad psychological and experiential views of mental health ( *e.g.*, Johnstone & Boyle, 2018), as well as more traditional diagnostic models of mental health conditions ( Larsen & Hastings, 2018). An ontology can help organise and synthesise data from various sources (e.g., findings from studies informed by different views of mental health). This aspect of ontological frameworks can be particularly useful for the mental health domain, considering ongoing debates about the best way of classifying mental health conditions. For example, key debates have centred around people with the same diagnoses experiencing very different symptoms, while people with different diagnoses experiencing the same or very similar symptoms ( Clark *et al.*, 2017; Conway *et al.*, 2021; Feczko *et al.*, 2019; Robinaugh *et al.*, 2020). Given these debates, the ontology aims to provide a strategy for representing experienced symptoms as entities in addition to representing diagnoses and the potential interrelationships between these. Mental health classification systems such as DSM-5, ICD and RDoC ( Clark *et al.*, 2017) can be explicitly supported through cross-references to relevant ontological entities.

As the current ontology is developed to organise data relevant to systematic reviews about anxiety, depression and psychosis in the GALENOS Project’s data repository, this ontology will focus on these three mental health conditions. However, the upper-level structure will be broad enough to be relevant to any mental health condition (as well as to broadly capture any population or intervention). Therefore, the upper-level entities and structure can serve as a foundation for developing an ontology of mental health beyond the current scope. A key advantage of ontologies is that they can be continually updated based on evidence and feedback ( Arp *et al.*, 2015; He *et al.*, 2018), allowing entities and their relationships to evolve in response to broadening consensus within the mental health field.

Ontologies are ‘readable’ by both humans and computers and therefore can be used to generate computer algorithms to categorise, retrieve and synthesise evidence ( Hastings, 2017; Seppälä *et al*., 2014). Within the GALENOS repository, the ontology will enable linking and synthesising data across reviews. As more research enters the system and is classified according to concepts in the ontology, machine learning will become more attuned to the precise research relevant to each living systematic review. We will use the ontology to populate a comprehensive online living evidence summary (see AD-SOLES, for example) ( Hair, 2022).

The GALENOS Mental Health Ontology will:

- provide a shared framework to communicate, organise and analyse evidence about aspects of anxiety, depression and psychosis across research teams in GALENOS Project;
- support the online data repository by informing how research is browsed, categorised, indexed and summarised;
- facilitate the use of machine learning algorithms as a step towards enabling more efficient processes to categorise, retrieve and synthesise evidence as it is published in the future;
- provide upper-level entities and structure to serve as a foundation for developing an extensive ontology of mental health.

## Methods

### Set up the Mental Health Ontology Advisory Board

***Terms of reference of Advisory Board***

Members of the advisory board will bring their perspectives to the work, recognising that ontologies seek to reflect many perspectives, and that consensus is aimed for but not always immediately achieved ( Open Biological and Biomedical Ontology [OBO] Foundry, 2019). They will be invited to attend online meetings to provide feedback during ontology development about the methodology, emerging ontology content and organisation, and ontology-structured evidence. They will also be invited to submit feedback to written documents that will inform ontology development. Based on the number of participants in previous studies to provide feedback on ontologies, we aim to recruit at least 10 members for the advisory board before the initial round of feedback ( Michie *et al.*, 2020; Norris *et al.*, 2020). However, this number is subject to change, with more experts being recruited when people with relevant expertise and lived experience express interest and/or specific expertise are needed or available.

***Criteria for selection of members***

Selection to reflect representativeness across geography and discipline include:

1. Representation from Global Experiential Advisory Board
2. Volunteers from the Galenos International Advisory Board (including experts with animal and human science content expertise)
3. Individuals who have done work in Mental Health classification or measurement
4. Mental Health organisations to be invited to send a representative
5. Ontology experts

***Current members of the advisory board***

At the time of submission, the Mental Health Advisory Board has 17 board members from 10 countries (Australia, Belgium, Canada, Germany, India, Israel, Portugal, UK, USA and Zimbabwe), with expertise in various domains, including psychiatry, clinical psychology and health psychology, as well as lived experience.

### Ontology development and integration into the GALENOS Project repository

**Figure 2.**
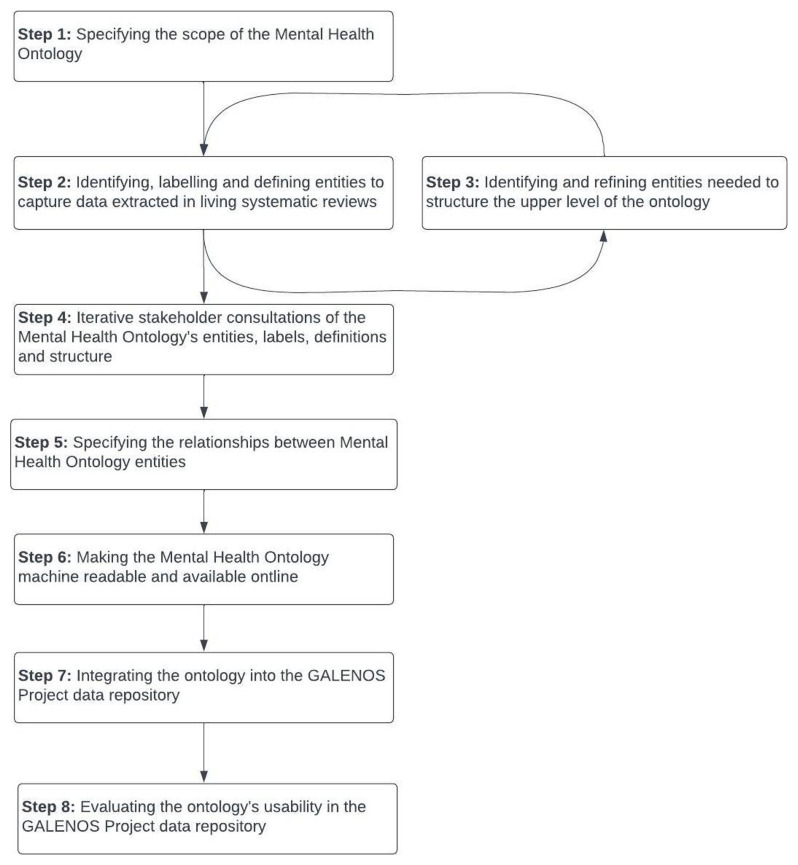
Overview of steps to develop the Mental Health Ontology.

The Mental Health Ontology will be developed and integrated into the GALENOS Project’s repository in eight iterative steps, broadly drawing on the methods applied for developing the Behaviour Change Intervention Ontology (BCIO; Wright *et al.*, 2020). Figure 2 presents an overview of these steps.

***Step 1: Specifying the scope of the Mental Health Ontology***

The preliminary scope of the Mental Health Ontology will cover: (1) human mental health conceptualisations, including constructs representing symptoms and conditions ( *i.e.*, diagnoses), (2) mental health interventions ( *i.e.*, coordinated sets of activities designed to change specified aspects of mental health) and their delivery, (3) settings in which interventions are delivered, (4) populations to whom interventions are delivered, (5) intervention mechanisms and biomarkers for mental health outcomes, (6) intervention outcomes (including risk prediction) and spillover effects related to mental health and (7) research methods. The ontology’s level of detail for entities will be informed by its use case in the GALENOS Project, namely integrating evidence across systematic reviews. Therefore, we will only include detailed entities where required for the associated data extraction of these reviews, focusing on anxiety, depression and psychosis. This scope will be refined during later steps.

***Step 2: Identifying, labelling and defining entities needed for living systematic reviews***

The GALENOS Project’s ontology development and systematic review teams will work together to identify and refine entities that will be used when extracting data and storing data as part of the living systematic reviews. Approximately three systematic reviews on human studies will be conducted per year, completing in January 2026. Each is led by two or three researchers with an MSc or PhD in an area related to mental health.

The review teams will share an initial extraction template with the ontology team, who will review these sheets and provide feedback about their clarity and propose potential ontological entities for defining constructs. To ensure that the ontology is fit for purpose for structuring the data in the data repository, the ontology development team will review the extracted data, once each systematic review is completed. These data will be reviewed in an Excel spreadsheet, and a researcher will annotate the categories for which data are extracted, using entities or developing new entities in the ontology.

To capture categories for which data were extracted in the Mental Health Ontology, the ontology development team will first check if a relevant entity is already included in the Mental Health Ontology. If not, the team will identify relevant entities from existing ontologies or develop new entities. Entities from other ontologies will be identified by using specialist ontology databases, *e.g.*, the Ontology Lookup Service ( European Bioinformatics Institute, 2019), and where appropriate, these entities will be reused or cross-referenced in the Mental Health Ontology. For example, we will reuse relevant parts from the Behaviour Change Intervention Ontology (BCIO; Michie *et al*., 2020), the Mental Functioning Ontology (MFO; Hastings *et al*., 2012), Emotion Ontology (MFOEM; Hastings *et al*., 2011), Information Artifact Ontology (IAO; Ceusters & Smith, 2015). Where we identify that changes to an external ontology are needed, we will log the relevant change on the external ontology’s GitHub repository. New entities will be developed, labelled and defined by drawing on mental health classification systems or dictionaries, and assigned unique alphanumeric identifiers.

To avoid introducing too much detail into the ontology, detailed categories will be annotated using multiple entities to capture all aspects of these categories. For example, the category ‘mean anhedonia at follow up’ could be captured with the entities ‘average value’, ‘anhedonia symptom severity’ and 'measurement datum at followup’.

**Figure 3.**
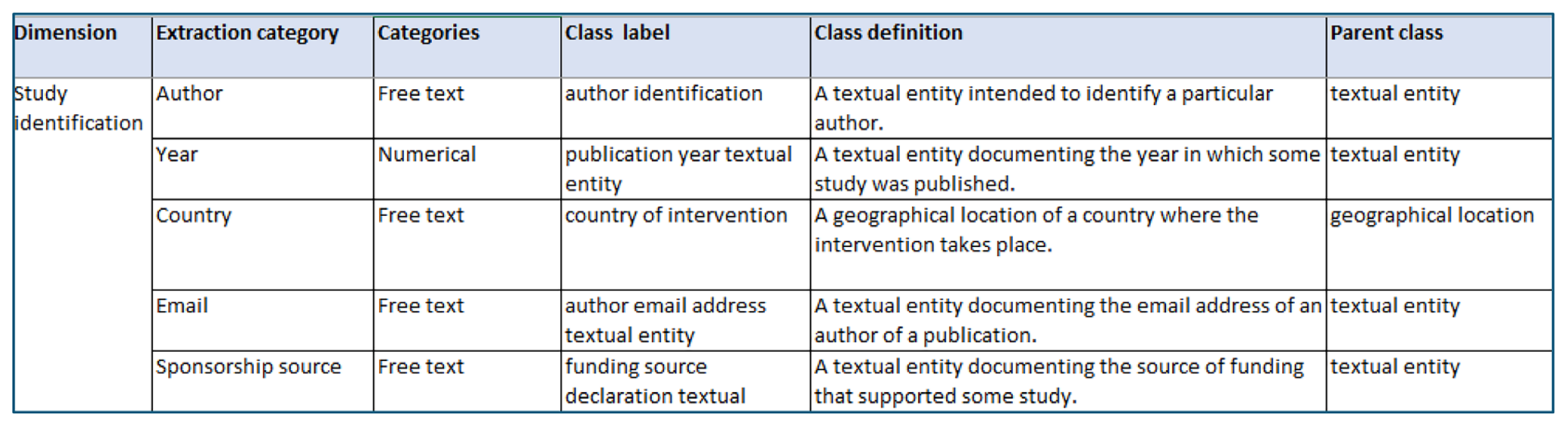
Example of mapping ontology entities onto extraction sheet used in a systematic review.

Once categories in the data extraction sheets have been annotated with ontological entities, the ontology development team will share these entities, their labels and definitions with the systematic review teams. These teams will provide feedback on whether each entity appropriately captures the category of interest (see example mapping presented to review teams in Figure 3). Where teams suggest changes to entities, updates will be made to the ontology and annotation record where necessary. This annotation record will be used to inform the structure of the data repository. In addition, if the entities have previously been applied by other systematic review teams, they will be informed of the changes and given the opportunity to raise issues with these changes.

***Step 3: Identifying and refining entities needed to structure the upper level of the ontology***

Parallel to developing entities used to map the living systematic review, we will identify upper-level entities (e.g., ‘mental health intervention’) to provide the Mental Health Ontology an overarching structure. Such a structure can help organise the entities in the ontology, as well as serve as a scaffold to build a more comprehensive ontology for the mental health field in the future.

To identify the upper-level entities, we will first review the upper-level entities in the BCIO ( Michie *et al*., 2020) and note down entities (e.g., ‘intervention population’) that are relevant to mental health and mental health interventions. The BCIO was selected as starting point, as the mental health ontology will need to synthesise data from interventions. Relevant BCIO entities (e.g., ‘behaviour change intervention’) can be used as examples to inform the development of corresponding entities needed for the current scope, namely mental health (e.g., ‘mental health intervention’). Some GALENOS systematic reviews will have research questions about human populations beyond interventions (e.g., the effect of childhood experiences). Therefore, the structure of the Mental Health Ontology upper-level entities will be specified to represent knowledge about interventions, but also human populations more generally (e.g., representing that human populations may or may not participate in interventions). After the core ontology team has drafted these entities, they will be presented to the wider GALENOS team and ontology advisory board for feedback and updates will be made accordingly.

***Step 4: Iterative stakeholder consultations of the Mental Health Ontology***

Stakeholder consultations will be conducted to refine (1) the labels and definitions of entities that will be used in the GALENOS data repository and (2) the upper-level entities of the ontology. For the entity labels and definitions that will be applied in the GALENOS repository, a stakeholder consultation will inform the development of the repository to enable it to be comprehensible and usable by people interested in understanding and engaging with the data (e.g., researchers interested in reusing the data). In parallel, the consultation on the upper-level entities aims to ensure that the ontology’s broader structure: (1) clearly reflects broad entities important to people experiencing mental health issues, (2) captures a broad scientific consensus in the mental health field, and (3) meets the needs of potential ontology users ( OBO Foundry, 2019; Wright *et al.*, 2020).

Participants will be recruited by (1) inviting members of the Mental Health Ontology Advisory Board,(2) asking these members to suggest individuals or groups with relevant expertise, and (3) advertising the study through the UCL Centre of Behaviour Change and GALENOS Project’s official social media accounts (LinkedIn and X). The consultations will be carried out via Qualtrics surveys and, before each consultation, participants will be provided with general training that covers: (1) what an ontology is and (2) an overview of the Mental Health Ontology.

When developing the materials for the stakeholder consultations, we will ask for feedback from at least one experiential advisor member of the Mental Health Ontology Advisory Board in order to enable the participation of people with lived experience of mental health issues in the stakeholder consultation process ( National Institute for Health Research [NIHR], 2019).

**Stakeholder consultations on entities used for the GALENOS data repository**

For every set of three systematic reviews conducted annually, we will carry out a stakeholder consultation, in which new entity labels and definitions will be reviewed for clarity. We aim for each entity to be reviewed by at least 10 participants, which aligns with the number of participants considered appropriate for stakeholder consultations (3–29 participants) as part of the BCIO ( Michie *et al.*, 2020; Norris *et al.*, 2020). Only entities needed for the repository will be included in these consultations. To allow diverse participants to provide feedback and limit the burden on participants, the entities will be presented in sets of around 40 entities, keeping the time to complete the surveys between 45–60 minutes.

In the survey, participants will be presented with a label and definition for each entity, and asked:

1). Whether any entity, label or definition is unclear
2). Whether an everyday English (informal definition) is needed to support understanding the entity

Where participants indicate that an entity is unclear, they will be prompted to suggest improvements to the entity. At the end of the survey, participants will be asked to provide any feedback that was not prompted by other questions.

**Stakeholder consultation on upper-level entities**

We aim to recruit at least 10 participants per stakeholder review, with broad theoretical knowledge and expertise relating the mental health field or lived experience of mental health conditions or expertise relating to ontologies. The number of participants is considered appropriate based on the development of ontologies part of the BCIO, which included 3–29 participants in their stakeholder consultations.

In a survey, they will then be presented with: (1) the Mental Health Ontology’s upper-level entities and relationships as a diagram, (2) a list of the relationships between entities, which are informally described to help participants better understand these relationships and (3) the labels an definitions of the upper-level entities. Participants will be invited to provide feedback on the upper-level entities and relationships in the ontology in terms of:

1). The clarity of the upper level: Whether any entity, label or definition is unclear
2). The comprehensiveness of the upper level: Whether any entities are missing from the upper level of the ontology
3) Accuracy of relationships: Whether any relationships between entities need to be changed to better capture aspects of mental health

Where participants indicate that entities are unclear or missing, they will be asked to suggest relevant changes to improve the ontology. Participants will also be asked if they have any additional feedback which was not prompted by other survey questions.

**Analysis of stakeholder consultations**

Each piece of feedback from the participants will be recorded and reviewed by a researcher (PS or MS) to propose changes to the ontology. The relevant feedback and proposed changes will be discussed among the researchers leading the Mental Health Ontology’s development (JH, MS, SM & PS). We will record decisions regarding how each piece of feedback will be addressed. To verify that appropriate changes have been made to entities that will be used in the repository, updated entities will be shared and reviewed by researchers leading the relevant living systematic review. The updated upper level of the ontology will be presented to the ontology advisory board to verify that the changes to the ontology are appropriate to both academic experts and experiential advisors in mental health.

***Step 5: Specifying the relationships between Mental Health Ontology entities***

The ontology development team will discuss, specify and refine the relationships between entities in the Mental Health Ontology. Common relationships ( *e.g.*, ‘is_a’ and ‘has_part’) will be used from the widely used upper-level ontologies ***Basic Formal Ontology*** and the Relation Ontology ( Smith *et al.*, 2005). To structure the ontology, each entity will be linked to a ***parent class*** with a hierarchical ‘is_a’ relationship ( Arp *et al.*, 2015; Smith *et al.*, 2005). For instance, the entity ‘motivation’ will have an ‘is_a’ relationship to its parent class ‘mental process’: motivation ‘is_a’ mental process. The team will also discuss whether any new relations need to be specified between entities to structure the ontology and, if so, develop such relations.

***Step 6: Making the Mental Health Ontology machine-readable and available online***

We will develop the Mental Health Ontology as a spreadsheet of entities: Each entity will be organised as a separate row with a primary label and definition, unique alphanumeric identifier ( *i.e.*, ***Uniform Resource Identifier [URI]*** ; e.g., BCIO:01023), relationships, and if available, synonyms, informal definitions and examples. These fields ( *e.g.*, label and definition) will be organised into separate columns. When the ontology’s content is ready for its initial release, we will convert this content to ***Web Ontology Language (OWL)*** ( Antoniou & van Harmelen, 2004) format. In this standard format, the ontology can be viewed and visualised within ontology software, such as Protégé ( Musen, 2015), and becomes compatible with other ontologies. For the conversion to OWL, we will use the ***ROBOT*** ontology toolkit library ( Jackson *et al.*, 2019), which supports creating well-formatted ontologies from spreadsheet-format templates. The ROBOT template is a comma-separated values (CSV) file that is prepared from the primary ontology spreadsheets by adding instructions to the template header about how spreadsheet columns are to be converted into OWL and metadata attributes. The Mental Health Ontology’s OWL version will be stored on the ***GitHub*** repository of the project, as this repository supports versioning of the ontology, *i.e.*, it keeps a record of different versions of the ontology and any updates made. GitHub also has an ***issue tracker*** that enables ontology users to submit any issues with the ontology and ontology developers to respond to these issues.

***Step 7: Integrating the ontology into the GALENOS Project data repository***

After releasing the ontology through GitHub, we will closely collaborate with the GALENOS team who are developing the online data repository. We will add the mapping of the ontology onto the extracted data categories for each systematic review (see Step 2.2) as a CSV file to GitHub. This mapping, along with the ontology in its OWL format, will be shared with the data repository team. The data repository team will integrate the ontology’s structure and the mapping of entities onto the relevant systematic review on https://galenos-data.aliveevidence.org/. In the data repository, the ontology’s key application will be that entity labels and definitions will appear when hovering over relevant data categories for each living systematic reviews. In the backend, the same entities which have been mapped onto different systematic reviews will be linked, allowing searchability and integration across reviews.

***Step 8: Evaluating the ontology's usability in the GALENOS Project data repository***

The data repository’s usability, along with the clarity of the ontology mapping, will be evaluated through feedback from potential users. We will recruit participants through the GALENOS Project’s contacts, including the advisory boards, and official social media accounts (LinkedIn and X). Participants will be given prompts to engage with the data based on their interests (e.g., finding a living systematic review, and data on a specific category, such as mean age). We will use think-aloud methods to explore the usability and acceptability of the repository interface ( McDonald *et al*., 2012; Peute *et al*., 2015), including whether entity labels and definitions used for the systematic reviews are sufficiently clear. These sessions will be recorded, and analysed using thematic analysis to identify aspects of the repository that are usable and acceptable and aspects that are not in order to inform improvements to this repository ( Crane *et al*., 2017). The GALENOS teams will make updates to the repository interface and, where needed, the ontology.

### Applying the ontology to develop tools for data searching, visualising, extraction and synthesis, and partial automation of these processes

The Mental Health Ontology will be used for annotations of the ‘living evidence’ extracted from the literature and stored in the project’s online data repository. The systematic review publications will be linked to this database, and will be regularly updated, allowing new data to be retrieved and displayed ( *e.g.*, as plots) as part of the living systematic review. The ontology will also be used to develop tools and algorithms to support interoperability with other knowledge resources, enhanced searching, browsing and navigating of the evidence database, and ontology-based summarising and visualising the data. In conjunction with machine learning algorithms and the data from living systematic reviews carried out early in the GALENOS Project’s lifecycle, the ontology will also be applied to develop and test structured search strategies for later systematic reviews. The algorithms developed will be informed by the evolving deliverables and needs of the GALENOS Project. Thereby, the ontology development team aims to deliver:

1. A mental health ontology that is interoperable to enable more discoverable and translatable evidence across various sources, including early phase and late phase trials
2. Ontology-based algorithms to enable evidence searching, visualisation and querying
3. An open, coded and queryable database of relevant studies, characteristics of studies, risk of bias assessments and results data, richly linked to ontologies for interoperability

## Ethics

Ethical approval was granted by University College London’s ethics committee (CEHP/2020/579) in 2020. Participant informed written consent will be sought at the beginning of each stakeholder consultation.

## Study status

We have invited participants to join the Mental Health Advisory Board and organised the first advisory board meeting, specified the initial scope of the ontology (Step 1), drafted the initial entities for the first three systematic reviews as part of the GALENOS Project for the extraction sheets and revised these with input from systematic review teams (Step 2), drafted the first version of the upper-level entities and specified their relationships (Step 3) and started data collection for the stakeholder consultation about the ontology's upper level (Step 4).

## Conclusion

The Mental Health Ontology will be developed to serve as a shared framework to categorise, label and define entities relating to anxiety, depression and psychosis research within the GALENOS Project. The entities will include key constructs for diagnoses of conditions affecting mental health, experiences related to mental health, mental health interventions, their target populations and settings, intervention mechanisms and biomarkers for mental health outcomes, intervention outcomes and research methods. As these groups of constructs will each be elaborated for the domains of anxiety, depression and psychosis, and categorised in the ontology, it will enable the representation of entities relevant to different perspectives about research in these three domains and the integration of evidence from sources informed by such perspectives.

This ontology will be used to support structuring the GALENOS data repository, and thereby linking, integrating, analysing and visualising data. We will develop this ontology iteratively, updating it based on the needs of living systematic reviews and stakeholder feedback. As ontologies are computer readable, some of these processes can also be partially automated in the project lifecycle or refined to be fully automated after the project.

The Mental Health Ontology will be developed and maintained as part of the GALENOS Project, but beyond this project, the ontology will also be maintained alongside the BCIO as part of a 5-year long US National Institutes of Health (NIH) grant ( https://reporter.nih.gov/project-details/10938088). During this time, any issues on the ontology that are reported on GitHub will be tracked, responded to and, where needed, addressed by updating the ontology. In the future, this ontology, especially its upper level, has the potential to be expanded to capture mental health conditions beyond anxiety, depression and psychosis. For example, the ontology’s upper-level structure could be applied to broadly organise information about various mental health conditions across different categorisation systems and create new lower-level entities to capture aspects of mental health (e.g., about populations and interventions), cross-referencing the relevant categorisation systems.

## Data Availability

No data are associated with this article.
