# Supplementary material for: Towards an ontology of mental health: Protocol for developing an ontology to structure and integrate evidence regarding anxiety, depression and psychosis
Source: Wellcome Open Res. 2025 Aug 28;9:40. Originally published 2024 Feb 15. [Version 3] doi: 10.12688/wellcomeopenres.20701.3 (PMC11729156; doi:10.12688/wellcomeopenres.20701.3)
Supplement: Peer review history

# Reviewer report: Reviewer response for version 3

**DOI:** 10.21956/wellcomeopenres.27402.r130880

**Published:** 2025-11-03

**Authors:** Lilith Abrahamyan Empson

**Affiliations:** 1 Faculty of Biology and Medicine, Lausanne University Hospital and University of Lausanne, Lausanne, Switzerland

No further comments

Is the study design appropriate for the research question?

Partly

Is the rationale for, and objectives of, the study clearly described?

Yes

Are sufficient details of the methods provided to allow replication by others?

Partly

Are the datasets clearly presented in a useable and accessible format?

Not applicable

Reviewer Expertise:

Early Intervention in Psychiatry, early psychosis, urban mental health, participatory methodologies.

I confirm that I have read this submission and believe that I have an appropriate level of expertise to confirm that it is of an acceptable scientific standard.

# Reviewer report: Reviewer response for version 3

**DOI:** 10.21956/wellcomeopenres.27402.r130881

**Published:** 2025-09-17

**Authors:** Cecilie Thøgersen-Ntoumani, Bibi Dige Heiberg, Kristina Pfeffer, Chiara Cimenti

**Affiliations:** 1 Danish Centre for Motivation and Behaviour Science (DRIVEN), Department of Sports Science and Clinical Biomechanics, University of Southern Denmark, Odense, Denmark; 2 Department of Sports Science and Clinical Biomechanics, The Faculty of Health Sciences , Research Unit of Clinical Biomechanics, Odense, Denmark; 3 University of Southern Denmark, Odense, Denmark

We all are happy with the revisions the author made.

Is the study design appropriate for the research question?

Yes

Is the rationale for, and objectives of, the study clearly described?

Yes

Are sufficient details of the methods provided to allow replication by others?

Partly

Are the datasets clearly presented in a useable and accessible format?

Not applicable

Reviewer Expertise:

Fidelity, Musculoskeletal health, participatory approaches, stakeholder involvement, Biopsychosocial paradigm, motivation, behavioural psychology

We confirm that we have read this submission and believe that we have an appropriate level of expertise to confirm that it is of an acceptable scientific standard.

# Reviewer report: Reviewer response for version 2

**DOI:** 10.21956/wellcomeopenres.25788.r116802

**Published:** 2025-02-19

**Authors:** Lilith Abrahamyan Empson

**Affiliations:** 1 Faculty of Biology and Medicine, Lausanne University Hospital and University of Lausanne, Lausanne, Switzerland

Thank you for the opportunity to review this interesting and timely paper, which treats an important topic with possible clinical implications: create a comprehensive ontology on psychosis, anxiety and depression to better reflecting the diversity and complexity behind definitions, establishing links between them and providing with useful resources regarding clinical implications. 

 The quality of this work can be further improved on following aspects.

 1. Better definition of the perimeter in which this initiative can be usefully implemented in regards with cultural contexts and use of different classification systems. Indeed, stakeholders come from different geographical areas, which poses new methodological challenges. While stakeholders may use western classification systems, not all of them share the same cultural beliefs and practices and it is unclear whether and how these elements are taken into consideration? The same goes for the language and representativity of published materials that is going to be used. This is of high importance, especially in a endeavours that plan to include persons with lived experience, let alone professionals that are carriers of different cultures and languages. 

 In addition, some sentences may be misleading, such as '' to capture any population and intervention''. This may lead the reader think that a universality or a complete representativity is a given, yet the initiative is based only on western classification systems and paradigms, leaving out other classification systems and entire geographic zones, such as East Asia, Southeast Asia etc. 

 While it practically is impossible to include everyone ant take into account absolutely everything, it is important to acknowledge this limitations and be as clear and transparent about them. 

 2. Advisory board composition remains blurry and it remains unclear how representativity will be assured. The same goes for stakeholders. 

 3. While the inclusion of persons with lived experience is both commendable and necessary, it remains unclear what framework will be put in place to assure true participation. It is often a big challenge, when it comes to transdisciplinary collaborations and making sure that environments conducive to making everyone’s voice be heard it is generally useful to refer to some collaboration framework or guiding principles of user-inclusion. In addition, the precision '' at least one experiential advisor'' raises questions: is such a low number enough to bring the input needed. 

 4. Regarding living systematic reviews, it will be important to specify how extracted information will be organised and further utilised? Authors mention 3 parallel reviews and excel spreadsheets with annotations. But how those annotations will be structured? What analytical framework is going to be used to assure that three parallel reviews will obey the same logic? At which point qualitative analysis will be conducted and how it will be driven?

Is the study design appropriate for the research question?

Partly

Is the rationale for, and objectives of, the study clearly described?

Yes

Are sufficient details of the methods provided to allow replication by others?

Partly

Are the datasets clearly presented in a useable and accessible format?

Not applicable

Reviewer Expertise:

Early Intervention in Psychiatry, early psychosis, urban mental health, participatory methodologies.

I confirm that I have read this submission and believe that I have an appropriate level of expertise to confirm that it is of an acceptable scientific standard, however I have significant reservations, as outlined above.

# Response

**Published:** 2025-08-22

**Authors:** Paulina Margarete Schenk

**Affiliations:** University College London, London, England, UK

Thank you for the opportunity to review this interesting and timely paper, which treats an important topic with possible clinical implications: create a comprehensive ontology on psychosis, anxiety and depression to better reflecting the diversity and complexity behind definitions, establishing links between them and providing with useful resources regarding clinical implications. **Response:**  We thank the reviewer for their time in engaging with the protocol and providing valuable feedback for improving this work. 

 The quality of this work can be further improved on following aspects.

 1. Better definition of the perimeter in which this initiative can be usefully implemented in regards with cultural contexts and use of different classification systems. Indeed, stakeholders come from different geographical areas, which poses new methodological challenges. While stakeholders may use western classification systems, not all of them share the same cultural beliefs and practices and it is unclear whether and how these elements are taken into consideration? **Response:** We appreciate the comment and agree that there are likely to be various cultural differences and practices that stakeholders reflect in their feedback. With the upper-level entities, the intention is to provide a formal structure that includes categories beyond any specific classification system, for example, capturing ‘people’ and ‘mental health interventions’ as entities and specifying relevant relationships (e.g., some people participating in mental health interventions). The labels and definitions of these entities are intended to be broad and inclusive. Therefore, feedback from stakeholders that suggests the need to expand the entities to fit wider cultural contexts and practices will be used to: (1) make such entities more inclusive or (2) add relevant entities to more clearly capture additional aspects of mental health that need to be represented in the ontology. This has now been more explicitly specified in Step 4 under ‘Analysis of stakeholder consultations’:

- “ *To ensure that the ontology’s upper level is relevant to a range of geographical and social contexts, we will update the entities and their structure to be as inclusive as possible, informed by stakeholder feedback.* *For example, this may involve updating entity labels and definitions to be broader, allowing them to capture wider contexts, or adding specific entities to better represent aspects of mental health that were previously insufficiently covered.”*

The more detailed entities in the ontology will be informed by the needs of the living systematic reviews within the GALENOS Project, namely the specific categories or variables for which data is extracted within the reviews. The research questions of these reviews are informed by a Global Advisory Board part of the wider project, who explicitly discuss potential cultural beliefs and practices beyond western contexts. Lived experience advisors are also part of the systematic reviews and provide input at various stages, including the decisions about data extraction. Therefore, we expect the categories for which we generate the more detailed entities to have already gone through wider consultations. In the usability evaluation for the detailed entities used in the online repository (Step 8), we also aim to include participants from a range of backgrounds, including diverse geographies. This has now been more clearly specified in the protocol:

- “ *To ensure that we include participants from a range of backgrounds, we will ask the lived experience advisors of the GALENOS Project to circulate invitations to their networks*.”

It should be noted that we will avoid drawing on specific classification systems for the current version of the ontology, with concepts such as ‘diagnosis’ being defined as a label assigned to people based on specific criteria but not linked to any specific preferred classification. We appreciate that cultural and social differences in mental health contexts and literature will not be comprehensively addressed in the current work; however, we will attempt to be as inclusive as possible. We have also updated the ‘Conclusion’ section to note that further work will be needed to develop a more extensive ontology that reflects and appropriately integrates a wider range of cultural and social perspectives on mental health:

- “ *Further work, including wider application and feedback on the ontology, are needed to ensure that the ontology better reflects the complexity of different social and cultural perspectives of mental health and relevant interventions*.”

  The same goes for the language and representativity of published materials that is going to be used. This is of high importance, especially in a endeavours that plan to include persons with lived experience, let alone professionals that are carriers of different cultures and languages. **Response:** We appreciate the important point raised. The representativeness of published materials within the living systematic reviews is beyond the scope of the work on developing the ontology. However, as noted above, the Global Advisory Board of the wider GALENOS Project is involved at various decision-making stages of the living systematic reviews and so can support a more inclusive approach in formulating the research process and the included publications. Within ontologies, the formal language used can sometimes be challenging to understand. We plan to add more ‘informal definitions’ to increase the understanding of the entities in the ontology. For the online repository (structured using the ontology), which will include data from various systematic reviews, we will use ‘understandable labels’ to make the repository’s content more accessible to users. Details on the work to structure the repository have been added to the Methods section Step 7:

- “ *To ensure that the repository is presented in a usable format, the ontology’s formal structure will not be shown directly in the repository. Instead, discussions between the systematic review team (CF, JK & JP) and an ontology team member (MS) will inform how the upper levels in the repository should be structured. *For example, rather than using formal upper-level entities such as ‘disposition’ in the repository, the systematic review team may suggest presenting ‘population’ as the highest level in the repository. *The two teams will also collaborate on generating ‘understandable labels’ for each category extracted. The mapped ontology entities and their definitions (which will appear when hovering over the labels) will provide additional clarity*.”

The usability evaluation (Step 8) aims to identify how this repository and the language presented within it can be improved, see:

- “ *The GALENOS teams will make updates to the repository interface ( **e.g., improving the clarity of understandable labels)** and, where needed, the ontology*.”

In addition, some sentences may be misleading, such as '' to capture any population and intervention''. This may lead the reader think that a universality or a complete representativity is a given, yet the initiative is based only on western classification systems and paradigms, leaving out other classification systems and entire geographic zones, such as East Asia, Southeast Asia etc. 

 While it practically is impossible to include everyone ant take into account absolutely everything, it is important to acknowledge this limitations and be as clear and transparent about them. **Response: **The phrasing has been updated to be more precise, considering the reviewer’s valid concerns around the potential for a comprehensive and representative ontology:

- “… *as well as to **allow the inclusion of a wide range of populations or interventions in the future***”

Please note that classification systems can be linked to the Mental Health Ontology currently being developed, but this is not a key part of the current work. The relevant sentence in the Introduction has been updated to reflect this more clearly:

- “ *In the future, the ontology can be linked to existing mental health classification systems such as DSM-5, ICD and RDoC (* *Clark et al., 2017* *) by associating ontological entities with cross-references **to relevant DSM/ICD/RDoC concepts or categories (e.g., diagnosis)***.”

As indicated above, a sentence was also added to the ‘Conclusion’ section acknowledge that more work is needed for the ontology to reflect wider perspectives on mental health:

- “ *Further work, including wider application and feedback on the ontology, are needed to ensure that the ontology better reflects the complexity of different social and cultural perspectives of mental health and relevant interventions.”*

2. Advisory board composition remains blurry and it remains unclear how representativity will be assured. The same goes for stakeholders. **Response:** We added more details to provide an overview of the advisory board:

- “ *Many advisory board members have expertise in more than one discipline (e.g., clinical experience and health psychology research experience), with some focusing on specialised topics such as paediatric traumatic stress, pain and chronic illness, physical activity, mood disorders and various others. The members include 14 with professor, associate or assistant professor roles at universities (with some also working as clinicians), *a research fellow (JK) , a psychiatrist *and two lived experience advisors with undergraduate or post-graduate degrees related to psychology.*”

The inclusion criteria for the stakeholders have also been better specified:

- “ *The inclusion criteria will be having professional or volunteering experience on mental health project, being able to read and write in English and having access to an electronic device*.”

3. While the inclusion of persons with lived experience is both commendable and necessary, it remains unclear what framework will be put in place to assure true participation. It is often a big challenge, when it comes to transdisciplinary collaborations and making sure that environments conducive to making everyone’s voice be heard it is generally useful to refer to some collaboration framework or guiding principles of user-inclusion. In addition, the precision '' at least one experiential advisor'' raises questions: is such a low number enough to bring the input needed. **Response:** We agree with the reviewer that integrating lived experience is a challenge and warrants considerable attention. The GALENOS Project, as a whole, attempts to include lived experience advisors throughout the lifecycle of its systematic reviews, starting from the formulation of research questions to the publications of results. In the ontology work, we are planning to take a step in the right direction by finding points of entry in which lived experience advisors provide feedback on aspects of the ontology and the repository it informs. Two of the advisory board members of the ontology are lived experience advisors due to the availability of resources (see section ‘Step up the Mental Health Ontology Advisory Board’). Additional support has been offered to advisory board members, such as an additional introductory meeting for any informal questions on ontologies. While we acknowledge that having only one lived experience advisor review the materials may not be sufficient, we are also mindful that the advisors may not be available to engage at the same time. The wider advisory board members will also be invited to provide feedback to improve the materials. This is more clearly indicated now:

- “ *When developing the materials for the stakeholder consultations, we will ask for feedback **from the Mental Health Ontology Advisory Board** and at least one lived experience advisor in order to enable the participation of people* **less familiar with ontologies** *in the stakeholder consultation process ( National Institute for Health Research [NIHR], 2019*).”

To ensure a range of expertise are included in the consultation, the lived experience advisors will be encouraged to invite people with relevant lived experience to participate (see Step 4):

- *“… , asking these members to suggest individuals or groups with relevant expertise **, with the two lived experience advisors being asked separately to share the invitation with their networks **…”*

To ensure the inclusion of stakeholders with diverse experiences, we have also invited participants with professional or volunteering experience in mental health projects, see the inclusion criteria specified as:

- “ *The inclusion criteria will be having professional or volunteering experience on mental health project, being able to read and write in English and having access to an electronic device.”*

For the usability study (Step 8), we will similarly ask the lived experience advisors of the GALENOS Project to share the study invite to their networks:

- “ *To ensure that we include participants from a range of backgrounds, we will ask the lived experience advisors of the GALENOS Project to circulate invitations to their networks.”*

We appreciate the reviewer’s comments on the need for more methodological considerations on how to engage stakeholders. Please note that due the complexity of ontologies and their formal computer-readable language in-depth stakeholder involvement has challenges. Especially lived experience advisor involvement is uncommon in the development of these formal tools. The current work presents efforts in the right direct in terms of trying to engage lived experience advisors to improve the formal language used about mental health. For example, for the current stakeholder consultation, we planned to show training videos to help participants familiarise themselves with ontologies and the content of the mental health ontology (see Step 4):

- “ *Before being invited to complete the survey, participants will be provided with online training videos that cover: (1) what an ontology is and (2) an overview of the Mental Health Ontology*.”

From this project, we also aim to learn how to engage stakeholders and lived experience advisors more effectively and thoroughly in the future.

 4. Regarding living systematic reviews, it will be important to specify how extracted information will be organised and further utilised? Authors mention 3 parallel reviews and excel spreadsheets with annotations. But how those annotations will be structured? What analytical framework is going to be used to assure that three parallel reviews will obey the same logic? At which point qualitative analysis will be conducted and how it will be driven? **Response:** Thank you for this note. To provide more information on the systematic reviews, a list of these reviews is now available in Table 2. To be clearer, we updated the section explaining the process of developing entities to capture the categories for which data had been extracted in the systematic reviews (see Step 2). This included adding an explanation of this step earlier on:

- “ *To ensure the ontology is fit for structuring the GALENOS online repository, ontological entities will be identified to capture the data extracted in the GALENOS Project's living systematic reviews. The project’s ontology development and systematic review teams will work together to identify and refine these ontological entities*.”

The step involves a mapping exercise in which ontology classes are mapped to (or annotated onto) the categories for which data is extracted. We now avoided using the term ‘annotation’ for the mapping process to reduce confusion with the annotations done during the systematic reviews. We also reformulated the more detailed paragraph describing this process to be clearer:

- “ *Once each review is completed, the ontology development team will review the data extracted within the systematic reviews from published papers (e.g., mean age and mental health outcomes) in an Excel spreadsheet. The team will focus on formally capturing the categories for which data is extracted (e.g., mean age) in the ontology, rather than capturing the entire extracted dataset (e.g., a study’s mean age being 45) in an ontological format. These categories (e.g., mean age) are captured through a mapping exercise, in which one or two researchers (MS & PS) map ontology entities onto these categories, developing new entities where needed (see example in Figure 3). *

*For the mapping exercise, the ontology development team will first check if a relevant entity is already included in the Mental Health Ontology…”* The annotation process within the systematic reviews will be informed by the extraction sheets that were developed by the relevant research team and through consultations with the GALENOS Global Advisory Board. As the reviewer notes, with different researcher leading the systematic reviews, there are challenges in systematising the data extractions. For this purpose, the project has now established a Data Governance team, including two members of the ontology development team (MS & JH) and members from the living systematic review teams (including the co-authors CF and JK). Details about this have been added to Step 2:

- “ *Following the first three systematic reviews, it became clear that stronger data governance was needed during the preparation and data extraction phases of the reviews. Therefore, members of the ontology development team (MS & JH), the living systematic review teams (CF, JK & SS) and the data repository team (DB) are meeting regularly to formulate rules for more consistent data extraction across different reviews.*”

The quantitative analysis for the systematic reviews is conducted separately. The current work aims to create a repository that provide a shared framework for organising data across the systematic reviews.

# Reviewer report: Reviewer response for version 2

**DOI:** 10.21956/wellcomeopenres.25788.r114717

**Published:** 2025-01-13

**Authors:** Cecilie Thøgersen-Ntoumani, Bibi Dige Heiberg, Kristina Pfeffer, Chiara Cimenti

**Affiliations:** 1 Danish Centre for Motivation and Behaviour Science (DRIVEN), Department of Sports Science and Clinical Biomechanics, University of Southern Denmark, Odense, Denmark; 2 Department of Sports Science and Clinical Biomechanics, The Faculty of Health Sciences , Research Unit of Clinical Biomechanics, Odense, Denmark; 3 University of Southern Denmark, Odense, Denmark

Thank you very much for the opportunity to review this interesting and important piece of work. In general, the authors have succeeded in clearly describing the complex process of developing this new ontology within the scope of mental health, presenting it in a clear and accessible way. This work aims to establish consistent terminology for mental health conditions, thereby enhancing behavioural science research in this area. It is clear that the initial round of reviews has generated many valuable revisions.

 We particularly appreciate the high degree of stakeholder involvement throughout the process and the attempt to visualize the process with the aid of figures. We also appreciate the list of definitions presented in Table 1 and throughout the text, which enhances the understanding of the methodology and background.

 We do, however, have some concerns that we recommend the authors to address, along with questions for clarification and suggestions that may be useful for future efforts in developing the ontology and refining the protocol.

**General comments:**

1. **Scope of mental health disorders**
  1.1. We believe that there are some incongruencies in the use of the term “mental health”, that require attention. The authors name the ontology a “mental health ontology” and define mental health as “a state of mental wellbeing...”. However, the reviews focus on three mental health conditions, which raise the question: should this ontology be defined as an ontology of mental health *conditions*? Furthermore, we wonder whether it can be defined as an ontology of mental health conditions when only three conditions are targeted. We would also suggest more justification as to why those three conditions have been chosen.
  1.2. We suggest adding clear definitions of the three chosen conditions as for example psychosis, can cover a range of different diagnoses with diverse symptoms (schizophrenia, schizoaffective disorders, bipolar disorder, substance-induced psychosis, post-partum etc.).

**2. Stakeholder involvement and leadership**

 We appreciate the attempt to make a thorough stakeholder involvement throughout the developmental process. We have the following suggestions to further enhance the stakeholder involvement :

  

- Leadership team: what is the distribution across clinicians, experts with lived experience, and researchers (from various fields)? And what counts as clinicians, experts with lived experience (i.e., definition)?
- How often approx. will the advisory board meet? And what will be the distribution across the various types of members?
- Stakeholder consultations: It is a bit unclear whether these are only survey-based? Is there any face-to-face consultation with the group? This is unclear and should be specified. Both would add value in different ways.
- It would be useful with a more iterative consultation phase, where, for example, participants with lived experience take part not in a single consultation but several over time, as these participants often need time to reflect on their lived experiences. An iterative process would allow for participants to refine and expand on their feedback and will therefore provide richer data. This process would foster more engagement, and more in-depth data.
- How will the research team manage disagreements between different stakeholders in terms of input into the ontology?
- “The updated upper level of the ontology will be presented to the ontology advisory board to verify that the changes to the ontology are appropriate to both academic experts and experiential advisors in mental health.” – how will potential discrepancies be handled to inform the ontology?

**3. Systematic reviews and ontology development**

- Step 2: it is stated “Approximately three systematic reviews on human studies will be conducted per year, completing in January 2026. Each is led by two or three researchers with an MSc or PhD in an area related to mental health.” How are the numbers of systematic reviews decided on? It would also be useful with some indication of the topics (indicative titles) for these systematic reviews.
  Please notice that we do not possess qualifications to review parts about repository building or machine learning.

**Specific comments:**

**Page 2**

**Plain language summary: **

- Why are 5 steps outlined here and not 8 steps as described in the methods section? Please align these.
- The plain language summary could be more “plain”/lay like. E.g., concepts, entities, classification frameworks are unlikely to be understood by a lay public.

**Page 4**

**1. The “piece by piece approach” to mental health** **research (Gardner & Kleinman, 2019): Mental** **health research**

- The phrasing “Piece by piece” could lead to the misunderstanding that this phrasing refers to a stepwise process more than a parallel process indicating development in silos. The authors could consider rephrasing the wording “piece-by-piece” – perhaps “piecemeal” approach would be clearer?
- In addition, the authors mention that research happens in silos in different fields. How will they ensure that this is not just a separate “behavioural science” silo and how will they facilitate the availability and use by others?

**4. Lack of focus on studying the mechanisms of mental** **health interventions**

- We are unsure whether "biomarkers" and "targets" are the most appropriate outputs for research about mechanisms. We suggest either elaborating on the following “can provide evidence for biomarkers for pharmacological, and targets for psychological and social interventions and” or delete it in order not to be too narrow or misleading in the description.
- “…evaluating existing evidence across animal and human data.” Why animal?
- “members from Canada, India, Nigeria, Philippines and Zimbabwe.” How come there are no members from Europe?

**Page 5**

- In the sentence starting with “In the GALENOS project, the….” you are referring to other ontologies. Can you specify exactly which ontologies you are referring to?
- In the sentence: “Constructs that overlap across systematic reviews can be identified and linked.”  Does this refer to linkage between various ontologies or reviews?

**Page 6:**

- It is a very positive and relevant feature that the upper-level structure will be broad enough to be relevant to any mental health condition.
- The following sentence is unclear: Mental health classification systems such as DSM-5, ICD and RDoC (Clark et al., 2017) can be explicitly supported through cross-references to relevant ontological entities.

**Page 7:**

- When stating “provide upper-level entities and structure to serve as a foundation for developing an extensive ontology of mental health.” Do the authors mean a new ontology on mental health in general?
- ***Step 1: Specifying the scope of the Mental Health Ontology***
  Regarding: “Therefore, we will only include detailed entities where required for the associated data extraction of these reviews, focusing on anxiety, depression and psychosis. This scope will be refined during later steps.” We believe it is very important to leave the ontology development open for future extensions because the described approach will by the nature of the process limit the amount of entities to the included reviews leaving out potential important aspects, especially regarding promising/evolving new areas within mental health research, that might not be captured by existing reviews.
- ***Step 2: Identifying, labelling and defining entities needed for living systematic reviews***
  Consider being more precise about the minimal criteria instead of a vague description, allowing for large variations in qualifications.
  When writing: “These data will be reviewed in an Excel spreadsheet, and a researcher will annotate the categories for which data are extracted, using entities or developing new entities in the ontology.” - Does this mean annotating scientific papers? If so, please specify.
- On page 7, in the first paragraph under Methods, the authors refer to online meetings where members of the Advisory Board will provide feedback. If we understand correctly, members of the Advisory Board will have the role of supervising the development of Ontology and providing their expertise, and stakeholders will include board members as well as other individuals. This could have been made clearer throughout the text as well as in Figure 2 concerning the role of both the Advisory Board and the stakeholder groups.

**Page 8**

**Figure 2. Overview of steps to develop the Mental Health Ontology.**

- This is a nice overview but is it possible to add details about stakeholder involvement - e.g. in different colours depending on the type/number of stakeholders and methods (survey/interview etc) for involvement.
- Step two continued)
  In which cases could the work on this ontology lead to relevant changes in other ontologies? This is a bit unclear to us.

**Page 9**

***Step 3: Identifying and refining entities needed to structure the upper level of the ontology***

- In general, we believe it is very important for the authors to ensure that the upper-level entities sufficiently broad
- Regarding presenting the first draft of the entities – Would it be feasible for the wider GALENOS team and advisory board to provide suggestions for upper-level entities before they are presented with the first draft. This to make sure that important aspects are not left out early in the process.

***Step 4: Iterative stakeholder consultations of the Mental Health*** ***Ontology***

- Can the authors specify stakeholder criteria?
- How will the authors ensure stakeholders are well represented in terms of education level, age and gender etc?
- Regarding stakeholder survey – consider presenting response options in the protocol paper.

**Pages 10-11**

- Step 8 (Evaluating the ontology): More information on the participants is required. Who are they? How many? Which groups will be represented?
- Do members of the GALENOS team overlap with the author team? Please specify
- It remains unclear how the researchers responsible for the development of the Mental Health Ontology´s will discuss the feedback and changes suggested by stakeholders (page 10, first paragraph under the heading “Analysis of stakeholder consultations”). This could be briefly clarified in one sentence with supporting references.

**Page 11**

- “Applying the ontology to develop tools for data searching, visualising, extraction and synthesis, and partial automation of these processes the Mental Health Ontology will be used for annotations of the ‘living evidence’ extracted from the literature and stored in the project’s online data repository.” It is unclear to us what this section means - especially regarding the living evidence. Can the authors please rephrase or clarify?

Is the study design appropriate for the research question?

Yes

Is the rationale for, and objectives of, the study clearly described?

Yes

Are sufficient details of the methods provided to allow replication by others?

Partly

Are the datasets clearly presented in a useable and accessible format?

Not applicable

Reviewer Expertise:

Fidelity, Musculoskeletal health, participatory approaches, stakeholder involvement, Biopsychosocial paradigm, motivation, behavioural psychology

We confirm that we have read this submission and believe that we have an appropriate level of expertise to confirm that it is of an acceptable scientific standard, however we have significant reservations, as outlined above.

# Response

**Published:** 2025-08-22

**Authors:** Paulina Margarete Schenk

**Affiliations:** University College London, London, England, UK

Thank you very much for the opportunity to review this interesting and important piece of work. In general, the authors have succeeded in clearly describing the complex process of developing this new ontology within the scope of mental health, presenting it in a clear and accessible way. This work aims to establish consistent terminology for mental health conditions, thereby enhancing behavioural science research in this area. It is clear that the initial round of reviews has generated many valuable revisions.

 We particularly appreciate the high degree of stakeholder involvement throughout the process and the attempt to visualize the process with the aid of figures. We also appreciate the list of definitions presented in Table 1 and throughout the text, which enhances the understanding of the methodology and background.

 We do, however, have some concerns that we recommend the authors to address, along with questions for clarification and suggestions that may be useful for future efforts in developing the ontology and refining the protocol. **Response:** We thank the reviewers for taking the time to review this protocol and providing valuable feedback to strengthen it. 

**General comments:**

1. **Scope of mental health disorders**
  1.1. We believe that there are some incongruencies in the use of the term “mental health”, that require attention. The authors name the ontology a “mental health ontology” and define mental health as “a state of mental wellbeing...”. However, the reviews focus on three mental health conditions, which raise the question: should this ontology be defined as an ontology of mental health *conditions*? Furthermore, we wonder whether it can be defined as an ontology of mental health conditions when only three conditions are targeted. We would also suggest more justification as to why those three conditions have been chosen.

**Response: **We appreciate the feedback. Based on the reviewer feedback in the first round, we would like to note that the protocol has been titled “Towards an ontology of mental health…” This was to emphasise that this work is not intended to develop an extensive ontology for all of the domain of mental health but to provide a starting point for such an ontology. As underlined in the protocol, we are aiming to develop an upper-level structure that helps capture evidence about mental health beyond the three conditions that the GALENOS Project focuses on. However, as the reviewers note, the detailed entities are developed to focus on aspects of anxiety, depression and psychosis as needed for the systematic reviews (see Step 2 of the Methods). On the second point regarding why this ontology is not called an ontology of mental health conditions, this phrase would restrict the intended scope to only the conditions themselves and associated symptomatology. However, the scope of the ontology encompasses protective factors and positive outcomes of mental health in general. Accordingly, the upper level of the ontology needs to accommodate positive aspects of mental health for the current use case and beyond, not only conditions and symptomatology. To make the scope of the ontology clearer, adjustments have been made to Step 1 of the Methods (see in bold):

- “ *The preliminary scope of the Mental Health Ontology will cover: (1) human mental health conceptualisations, including constructs representing symptoms, conditions ( i.e., diagnoses) **and wellbeing** **and*** ***promoting mental health rather than merely treating dysfunction with mental health*** *…”*
- “ *Therefore, we will only include detailed entities where required for the associated data extraction of these reviews, focusing on **research questions related to** anxiety, depression and psychosis.”*

For clarity, the title for Step 1 has also been updated to “Specifying the scope of the Mental Health Ontology **within the GALENOS Project**”, and the caption of Figure 2 has been updated to “Overview of steps to develop the Mental Health Ontology **within the GALENOS Project**”

 1.2. We suggest adding clear definitions of the three chosen conditions as for example psychosis, can cover a range of different diagnoses with diverse symptoms (schizophrenia, schizoaffective disorders, bipolar disorder, substance-induced psychosis, post-partum etc.). **Response:** We agree that the three conditions focused on, anxiety, depression and psychosis, cover a range of conditions and diagnoses with diverse symptoms. These conditions are among the top leading causes of global burden of ill mental health (GBD Collaborators, 2019); they were additionally prioritised as research in these areas can be ‘triangulated’ combining animal and human research to give more comprehensive insights into these areas (Cipriani et al., 2023). These terms are used as organising umbrella topics in the GALENOS Project, with the systematic reviews focusing on more specific topic areas. In most cases, more specific conditions, diagnosis or symptoms, such as social anxiety (Kennett et al., 2025), are investigated within the systematic reviews. We attempt to precisely capture these as labels assigned to people based on pre-specified criteria and, where relevant, we also capture entities for more specific symptoms. **2. Stakeholder involvement and leadership**

 We appreciate the attempt to make a thorough stakeholder involvement throughout the developmental process. We have the following suggestions to further enhance the stakeholder involvement:

  

- Leadership team: what is the distribution across clinicians, experts with lived experience, and researchers (from various fields)? And what counts as clinicians, experts with lived experience (i.e., definition)?

**Response:** The leadership described in the Introduction is for the GALENOS Project as a whole, rather than for the ontology work. As indicated in the Introduction, more details about these members can be found online on the GALENOS website: https://www.galenos.org.uk/about. We have added clearer signposting that information about contributors of the project can be found on the website:

- “These contributors include clinicians, researchers and lived experience advisors (mental health activists, campaigners and advocates), with expertise across data science, psychology and psychiatry. *Detailed information about the GALENOS Project **and its contributors** can be found on the project website (* *https://www.galenos.org.uk/about* *)...”*

As this leadership was not involved in the details of the current work, we did not add more information here. However, to provide a better overview of the researchers involved in the current work, we added the following information to the beginning of the Methods section:

- *“The development of ontology is being led by two researchers with post-graduate degrees in psychology and experience in mental health research (MS & PS). Two senior researchers, both with experience in ontologies and clinical psychology (JH & SM), supervise and regularly provide feedback on the ontology’s development and application. The project manager of GALENOS (JP) and two researchers leading systematic reviews within the project (CF & JK) also continuously support the work to refine the ontology and its application. Additional members across the GALENOS Project, such as from the data repository team (DB) and other systematic review team members (SS & SW), also regularly provide feedback to improve this work.”*

The lived experience advisors include mental health activists, campaigners and advocates from a range of countries (see https://www.galenos.org.uk/GLEAB). We added a clarification on this in the protocol: 

- *“These contributors include clinicians, researchers and lived experience advisors ( **mental health activists, campaigners and advocates**), with expertise across data science, psychology and psychiatry*.”

 

- How often approx. will the advisory board meet? And what will be the distribution across the various types of members?

**Response:** The advisory board meets 1-2 times a year, depending on the needs of the project. Details of this has been added to the Methods section “Set up the Mental Health Ontology Advisory Board”:

- “ *They will be invited to attend online meetings **once or twice a year, in which they will be given an overview of the methods and progress in developing the ontology. In these meetings, they will be prompted** to provide feedback about the methodology, emerging ontology content and organisation and ontology-structured evidence. They will also be invited to submit feedback to written documents that will inform ontology development **and join the formal stakeholder consultation to refine the ontology content (see Step 4) and usability evaluations of the ontology’s application (see Step 8).**”*

As various advisory board members work across interdisciplinary fields, an exact breakdown of their expertise is not possible. However, additional details have been added to provide a better overview of the board’s composition:

- *“Many advisory board members have expertise in more than one discipline (e.g., clinical experience and health psychology research experience), with some focusing on specialised topics such as paediatric traumatic stress, pain and chronic illness, physical activity, mood disorders and various others. The members include 14 with professor, associate or assistant professor roles at universities (with some also working as clinicians), *a research fellow (JK) *and two lived experience advisors with undergraduate or post-graduate degrees related to psychology.*”

 

- Stakeholder consultations: It is a bit unclear whether these are only survey-based? Is there any face-to-face consultation with the group? This is unclear and should be specified. Both would add value in different ways.

**Response:** The stakeholder consultations were planned to be primarily survey based. However, the methods planned for the upper level evolved to include a second round of feedback from the advisory board. Relevant sections in Step 4 have been updated to communicate this:

- *“This consultation will primarily be conducted through a Qualtrics survey, followed by a second round of feedback from advisory board members, involving an online meeting and then written input over email (see details in ‘Analysis of stakeholder consultations’).”*
- *“The updated upper level will be presented to the ontology advisory board **in a meeting** to verify that the changes to the ontology are appropriate to both academic experts and lived experience advisors in mental health. **Following the meeting, advisory board members will be invited to share additional comments via email to allow them more time to provide feedback on the changed entities. Drawing on these comments, any disagreements and potential updates will be discussed by the ontology development team, with a transparent log being kept showing how each comment was addressed**. *These logs, recording how each piece of feedback is addressed, will be shared on Open Science Framework. *”*

 It should also be noted that we adjusted the planned work of the stakeholder consultation to only focus on the upper level’s improvements. Instead, the usability work in the later stages will investigate the clarity and usefulness of the entities mapped to systematic reviews and therefore included in the repository. This was done to focus more on the usability of the ontology’s application within the repository, instead of conducting a separate stakeholder review for the entities that will be used in the repository (without the relevant context). The relevant sections were removed from Step 4. Instead, the usability section has been updated to more clearly reflect the work (see Step 8):

- *“In line with stakeholder consultations on ontologies (3–29 participants) as part of the BCIO (Michie et al., 2020; Norris et al., 2020) and relevant usability studies (Bruun & Stage, 2015), we will aim to recruit at least 10 participants. As the repository is likely to be used by people interested in data synthesis, the criteria will be for participants to have experience contributing to scoping or systematic reviews or applying review evidence in work related to mental health. To ensure that we include participants from a range of backgrounds, we will ask the lived experience advisors of the GALENOS Project to circulate invitations to their networks.*

*Participants will first be given an overview of the data repository by a researcher and then will be prompted to engage with the data in the repository based on their interests (e.g., finding a living systematic review, and data on a specific category, such as mean age). After participants have explored the repository on their own, a researcher will provide them with use cases about finding specific ~5 categories within the repository, visualising the data within these categories and engaging with the ontology classes mapped to the categories. To provide a wider range of different categories to engage with, the researchers will randomly select the ~5 categories from the full list of extracted categories for each participant. We will use think-aloud methods to explore the usability and acceptability of the repository interface (McDonald et al., 2012; Peute et al., 2015), including whether entity labels and definitions used for the systematic reviews are sufficiently clear. Following, the interviews, we will ask participants to fill out around 10 survey questions, adapted from the System Usability Scale (SUS) on the usability of digital systems (Brooke, 1996). The think aloud method and the survey questions are expected to take around 45-60 minutes to complete.”*  

- It would be useful with a more iterative consultation phase, where, for example, participants with lived experience take part not in a single consultation but several over time, as these participants often need time to reflect on their lived experiences. An iterative process would allow for participants to refine and expand on their feedback and will therefore provide richer data. This process would foster more engagement, and more in-depth data.

**Response:** We appreciate the suggestion for a more iterative consultation phase. We have adjusted the consultation of the upper level to include more iterative feedback on the updates from advisory board members (see Step 4: Iterative stakeholder consultations of the Mental Health Ontology):

- *“The updated upper level will be presented to the ontology advisory board **in a meeting** to verify that the changes to the ontology are appropriate to both academic experts and lived experience advisors in mental health. **Following the meeting, advisory board members will be invited to share additional comments via email to allow them more time to provide feedback on the changed entities. Drawing on these comments, any disagreements and potential updates will be discussed by the ontology development team, with a transparent log being kept showing how each comment was addressed**. *These logs, recording how each piece of feedback is addressed, will be shared on Open Science Framework. *”*

Unfortunately, the team does not have enough capacity to include iterative reviews for all parts of the ontology. Lived experience advisors, who are part of the project, have limited capability to be involved in the project as well, creating challenges for a more in-depth process than the proposed one. Please note that this section was updated from the previous round of review to make this work more realistic, and further adjustments needed to be made to focus more on the usability step (see Step 8), as indicated above.  

- How will the research team manage disagreements between different stakeholders in terms of input into the ontology?

**Response:** The descriptions in Step 4 have been updated to specify more clearly how disagreements will be addressed for each round of feedback:

- “ *In these discussions, the team will consider how the feedback will be addressed and review disagreements between stakeholder comments, documenting the rationale for implementing relevant changes in a log.”*
- “ *Following the meeting, advisory board members will be invited to share additional comments via email to allow them more time to provide feedback on the changed entities. Drawing on these comments, any disagreements and potential updates will be discussed by the ontology development team, with a transparent log being kept showing how each comment was addressed*.”

In response to another reviewer’s comments, we also added detail to more clearly communicate our aim to make the upper level as inclusive as possible:

- “ *To ensure that the ontology’s upper level captures a range of geographical and social contexts, we will aim to update the entities and their structure to be as inclusive as possible. Examples include updating entity labels and definitions to be broader to capture wider contexts or adding specific entities to better capture aspects of mental health that were previously insufficiently represented*.”

The final decisions about the updates to the ontology classes will be made by the research team. Decisions on the changes will be logged, along with the feedback received, to provide a transparent trail of the decisions made. The explanation of this log has been updated to explain that the relevant log will be included on OSF:

- “ *We will record decisions regarding how each piece of feedback will be addressed **, sharing this log on Open Science Framework***.”

 

- “The updated upper level of the ontology will be presented to the ontology advisory board to verify that the changes to the ontology are appropriate to both academic experts and experiential advisors in mental health.” – how will potential discrepancies be handled to inform the ontology?

**Response:** The following details have been added to the section on “Analysis of stakeholder consultation” to show how these comments are being addressed:

- “ *Following the meeting, advisory board members will be invited to share additional comments via email to allow them more time to provide feedback on the changed entities. Drawing on these comments, any disagreements and potential updates will be discussed by the ontology development team, with a transparent log being kept showing how each comment was addressed.”*

 
**3. Systematic reviews and ontology development**

- Step 2: it is stated “Approximately three systematic reviews on human studies will be conducted per year, completing in January 2026. Each is led by two or three researchers with an MSc or PhD in an area related to mental health.” How are the numbers of systematic reviews decided on? It would also be useful with some indication of the topics (indicative titles) for these systematic reviews.

**Response:** The number of the systematic reviews is guided by the GALENOS Project proposal and decided on by the leadership team. The plan was to generate about three systematic reviews per year, as this was deemed feasible given the capacity within the project. The ontology development responds to the needs of the project, integrating the evidence of the resulting systematic reviews. A table has now been added to provide an overview of the topics covered as part of the living systematic reviews planned (see Table 2). 

 Please notice that we do not possess qualifications to review parts about repository building or machine learning. **Response:** Thank you for noting this. 

**Specific comments:**

**Page 2**

**Plain language summary: **

- Why are 5 steps outlined here and not 8 steps as described in the methods section? Please align these.

**Response:** Thank you for the suggestion. We have updated the plain language summary to better align with the overall abstract; see the updates to the method specified within the plain language summary:

- “ *The ontology will be developed and applied by (1) specifying what it aims to cover, (2) identifying concepts that are needed in GALENOS Project’s systematic reviews (e.g., information on people’s ages and their diagnosis), (3) identifying broad concepts relating to mental health, such as ‘people’ or ‘mental health intervention’, that help organise more detailed concepts (e.g., ‘age’ organised as information relevant to people), (4) seeking  feedback from mental health experts and people with lived experience to improve the ontology, (5) linking concepts by specifying their relationships (e.g., ‘age’ is a characteristic of ‘people’), (6) making the ontology computer-readable and available online, (7) using the ontology to structure evidence from different systematic reviews in an online repository, and (8) evaluating whether this online repository is useable.*”

 

- The plain language summary could be more “plain”/lay like. E.g., concepts, entities, classification frameworks are unlikely to be understood by a lay public.

**Response:** We appreciate the feedback. In line with the suggestion, we have changed ‘concept’ to ‘category’. The term ‘entity’ was removed as it is not essential within the plain language summary. We changed the term ‘classification system’ to ‘categorisation system’.  We have attempted to simplify the language; the updated version attempts to balance the need to reflect the work simply, clearly and accurately. **Page 4**

**1. The “piece by piece approach” to mental health research (Gardner & Kleinman, 2019): Mental health research**

- The phrasing “Piece by piece” could lead to the misunderstanding that this phrasing refers to a stepwise process more than a parallel process indicating development in silos. The authors could consider rephrasing the wording “piece-by-piece” – perhaps “piecemeal” approach would be clearer?

**Response:** We changed the phrasing to “Silos in mental health research” in the Introduction (p. 4) to be clearer.    

- In addition, the authors mention that research happens in silos in different fields. How will they ensure that this is not just a separate “behavioural science” silo and how will they facilitate the availability and use by others?

**Response:** Ontologies should be designed to cross disciplinary boundaries and this is also the focus of the GALENOS Mental Health Ontology and the Behaviour Change Intervention Ontology. The current ontology will be aligned with the Behaviour Change Intervention Ontology, including some broad and overlapping entities (e.g., about interventions). However, the extent to which the Mental Health Ontology will be used across disciplines beyond the social and behavioural sciences will depend on dissemination and on providing tools and resources to facilitate this. The APRICOT (Advancing behavioural and social sciences through ontology tools) Project will help disseminate ontologies (including the Mental Health Ontology) and related tools across disciplines. A sentence reflecting this has been added to the Conclusion:

- “The Mental Health Ontology will be developed and maintained as part of the GALENOS Project, but beyond this project, the ontology will also be maintained alongside the BCIO as part of the **APRICOT (Advancing behavioural and social sciences through ontology tools) Project,** a 5-year long US National Institutes of Health (NIH) grant ( https://reporter.nih.gov/project-details/10938088). During this time, any issues on the ontology that are reported on GitHub will be tracked, responded to and, where needed, addressed by updating the ontology. **In addition, this project will support the dissemination of the ontology, introducing it to ontology developers and users interested in structuring knowledge about mental health across disciplines.”**

**4. Lack of focus on studying the mechanisms of mental health interventions**

- We are unsure whether "biomarkers" and "targets" are the most appropriate outputs for research about mechanisms. We suggest either elaborating on the following “can provide evidence for biomarkers for pharmacological, and targets for psychological and social interventions and” or delete it in order not to be too narrow or misleading in the description.

**Response:** The reference to ‘targets’ was made to more generally refer to mechanisms, which are targets within interventions. ‘Biomarkers’ are relevant within pharmacological interventions and relate to studying mechanisms, but we agree that the language around these could be clearer in the relevant point’s title.  In line with the current comment, we have updated the relevant title to read as follows (see bolded phrase for changes):

- “ *Lack of focus on studying the mechanisms **and biomarkers **within mental health interventions*”

 

- “…evaluating existing evidence across animal and human data.” Why animal?

**Response:** Evaluating and integrating evidence across animal and human data is part of the GALENOS Project’s overarching aims; these types of data produce evidence with different systematic errors and biases and triangulating them allows more comprehensive insights into the chosen topic area (Cipriani et al., 2023). However, as indicated in Step 1 (Specifying the scope of the GALENOS Mental Health Ontology), the ontology itself will focus on human data.  

- “members from Canada, India, Nigeria, Philippines and Zimbabwe.” How come there are no members from Europe?

**Response:** Members from Europe are already well represented in the leadership team (as indicated in the early part of the selected sentence). The aim of the Global Lived Experience Advisory Board is to integrate a wider range of perspectives into the decision-making process of the GALENOS Project as a whole.

**Page 5**

- In the sentence starting with “In the GALENOS project, the….” you are referring to other ontologies. Can you specify exactly which ontologies you are referring to?

**Response:** Thank you for the comment. The sentence is from the Introduction, so only a small edition has been made for clarity adding “where relevant”:

- “ *In the GALENOS Project, the ontological entities are developed or reused from other ontologies, **where relevant**, to organise constructs for which data are extracted in the systematic reviews*.”

We provide more details about the methods for identifying relevant ontologies in the Methods Section, including examples of the ontologies we are planning to draw on, please see in Step 2:

- *“Entities from other ontologies will be identified by using specialist ontology databases, e.g., the Ontology Lookup Service (* *European Bioinformatics Institute, 2019* *), and where appropriate, these entities will be reused or cross-referenced in the Mental Health Ontology. For example, we will reuse relevant parts from the Behaviour Change Intervention Ontology (BCIO;* *Michie et al., 2020* *), the Mental Functioning Ontology (MFO;* *Hastings et al., 2012* *), Emotion Ontology (MFOEM;* *Hastings et al., 2011* *) and Information Artifact Ontology (IAO;* *Ceusters & Smith, 2015* *).”*

 

- In the sentence: “Constructs that overlap across systematic reviews can be identified and linked.”  Does this refer to linkage between various ontologies or reviews?

**Response:** Thank you for noting this. The sentence has been updated to be clearer:

- “ *Constructs that overlap across systematic reviews can be identified and linked **by mapping these to the Mental Health Ontology and organising them in an online repository structured by the ontology**.*”

**Page 6:**

- It is a very positive and relevant feature that the upper-level structure will be broad enough to be relevant to any mental health condition.

**Response:** Thank you for the positive feedback on this. We have also updated the aims of the stakeholder consultation to more clearly indicate that we intend to be inclusive enough to capture positive and negative mental health experiences:

- *“This consultation on the upper-level entities aims to ensure that the ontology’s broader structure: (1) clearly reflects broad entities important to specify **people having positive or negative experiences related to their mental health**”*

 

- The following sentence is unclear: Mental health classification systems such as DSM-5, ICD and RDoC (Clark et al., 2017) can be explicitly supported through cross-references to relevant ontological entities.

**Response:** To be clearer, the relevant sentence has been changed to:

- **“ *In the future, the ontology can be linked to existing*** *mental health classification systems such as DSM-5, ICD and RDoC ( Clark et al., 2017) **by associating ontological entities with** cross-references **to relevant DSM/ICD/RDoC concepts or categories (e.g., diagnosis)**.*”

**Page 7:**

- When stating “provide upper-level entities and structure to serve as a foundation for developing an extensive ontology of mental health.” Do the authors mean a new ontology on mental health in general?

**Response:** The aim refers to the upper-level entities and structure can be used to expand the ontology to cover aspects of mental health more extensively in the future. It has been updated to be clearer:

- “… *provide upper-level entities and structure to serve as a **foundation for expanding this ontology into** an extensive ontology of mental health*.”

 

- ***Step 1: Specifying the scope of the Mental Health Ontology***
  Regarding: “Therefore, we will only include detailed entities where required for the associated data extraction of these reviews, focusing on anxiety, depression and psychosis. This scope will be refined during later steps.” We believe it is very important to leave the ontology development open for future extensions because the described approach will by the nature of the process limit the amount of entities to the included reviews leaving out potential important aspects, especially regarding promising/evolving new areas within mental health research, that might not be captured by existing reviews.

**Response:** The title for Step 1 has been updated to “Specifying the scope of the Mental Health Ontology **within the GALENOS Project**”. We need to reflect the scope to be realistic within the current project. As indicated, the detailed entities that will be added will focus on the application of the ontology, while the upper level of the Mental Health Ontology will accommodate the addition of entities on more varied topics in the future.  

- ***Step 2: Identifying, labelling and defining entities needed for living systematic reviews***
  Consider being more precise about the minimal criteria instead of a vague description, allowing for large variations in qualifications.
  When writing: “These data will be reviewed in an Excel spreadsheet, and a researcher will annotate the categories for which data are extracted, using entities or developing new entities in the ontology.” - Does this mean annotating scientific papers? If so, please specify.

**Response:** Thank you for the comment. The paragraph refers to the extraction templates that are used in the systematic reviews, which will be used to inform the entities in the ontology. To be clearer, several changes have been made to the Step 2 description. We described the purpose of this step earlier on:

- “ *To ensure the ontology is fit for structuring the GALENOS online repository, entities will be identified to capture the data extracted in the GALENOS Project's living systematic reviews. The project’s ontology development and systematic review teams will work together to identify and refine these ontological entities.”*

We also rephrased the paragraphs describing the mapping process to identify ontological entities to capture the categories for which data is extracted in the systematic reviews:

- “ *Once each review is completed, the ontology development team will review the data extracted within the systematic reviews from published papers (e.g., mean age and mental health outcomes) in an Excel spreadsheet. The team will focus on formally capturing the categories for which data is extracted (e.g., mean age) in the ontology, rather than capturing the entire extracted dataset (e.g., a study’s mean age being 45) in an ontological format. These categories (e.g., mean age) are captured through a mapping exercise, in which one or two researchers (MS & PS) map ontology entities onto these categories, developing new entities where needed (see example in Figure 3).*

*For the mapping exercise, the ontology development team will first check if a relevant entity is already included in the Mental Health Ontology….”*  

- On page 7, in the first paragraph under Methods, the authors refer to online meetings where members of the Advisory Board will provide feedback. If we understand correctly, members of the Advisory Board will have the role of supervising the development of Ontology and providing their expertise, and stakeholders will include board members as well as other individuals. This could have been made clearer throughout the text as well as in Figure 2 concerning the role of both the Advisory Board and the stakeholder groups.

**Response:** As the reviewer suggested, the Advisory Board will have a consulting role in providing feedback on the methods and development of the ontology. The stakeholder consultation will include advisory board members, but we will also make an effort to recruit a wider range of people to provide feedback. To be clearer, the details about the Advisory Board’s role and work has been updated to read as:

- “ *They will be invited to attend online meetings **once or twice a year, in which they will be given an overview of the methods and progress in developing the ontology. In these meetings, they will be prompted** to provide feedback about the methodology, emerging ontology content and organisation, and ontology-structured evidence. They will also be invited to submit feedback to written documents that will inform ontology **development and join formal stakeholder consultation to refine the ontology content (see Step 4) and usability evaluations of the ontology’s application (see Step 8).”***
- Figure 2 has been updated to illustrate who will be involved in the different steps.

 
**Page 8**

**Figure 2. Overview of steps to develop the Mental Health Ontology.**

- This is a nice overview but is it possible to add details about stakeholder involvement - e.g. in different colours depending on the type/number of stakeholders and methods (survey/interview etc) for involvement.

**Response:** Figure 2 has been updated to illustrate who will be involved in the different steps. However, we attempted to keep the figure simple. The details about the GALENOS members involved in the ontology’s development have been added under a new section titled " Developers and contributors to the ontology and its application in the GALENOS Project”:

- “ *The development of ontology is being led by two researchers with post-graduate degrees in psychology and experience in mental health research (MS & PS). Two senior researchers, both with experience in ontologies and clinical psychology (JH & SM), supervise and regularly provide feedback on the ontology’s development and application. The project manager of GALENOS (JP) and two researchers leading systematic reviews within the project (CF & JK) also continuously support the work to refine the ontology and its application. Additional members across the GALENOS Project, such as from the data repository team (DB) and other systematic review team members (SS & SW), also regularly provide feedback to improve this work.”*

The details about the Advisory Board’s involvement have been updated, as described above:

- “ *They will be invited to attend online meetings **once or twice a year, in which they will be given an overview of the methods and progress in developing the ontology. In these meetings, they will be prompted** to provide feedback about the methodology, emerging ontology content and organisation, and ontology-structured evidence. They will also be invited to submit feedback to written documents that will inform ontology **development and join formal stakeholder consultation to refine the ontology content (see Step 4) and usability evaluations of the ontology’s application (see Step 8)**.”*

Step 4 (the stakeholder consultations) and Step 8 (usability evaluation) have also been updated to provide an overview on the number of participants and methods earlier:

- “ *We aim to recruit at least 10 participants, with broad theoretical knowledge and expertise relating the mental health field, lived experience of mental health conditions or ontologies. The number of participants is considered appropriate based on the development of ontologies part of the BCIO, which included 3–29 participants in their stakeholder consultations. Participants will be recruited by (1) inviting members of the Mental Health Ontology Advisory Board, (2) *asking these members to suggest individuals or groups with relevant expertise, with the two lived experience advisors being asked separately to share the invitation with their networks *, and (3) advertising the study through the UCL Centre of Behaviour Change and GALENOS Project’s official social media accounts (LinkedIn and X). The inclusion criteria will be having professional or volunteering experience on mental health project, being able to read and write in English and having access to an electronic device.”* (Step 4)
- “ *In line with stakeholder consultations on ontologies (3–29 participants) as part of the BCIO ( Michie et al., 2020; Norris et al., 2020) and relevant usability studies (Bruun & Stage, 2015), we will aim to recruit at least 10 participants. As the repository is likely to be used by people interested in data synthesis, the criteria will be for participants to have experience contributing to scoping or systematic reviews or applying review evidence in work related to mental health. To ensure that we include participants from a range of backgrounds, we will ask the lived experience advisors of the GALENOS Project to circulate invitations to their networks.” *(Step 8)

 

- Step two continued)
  In which cases could the work on this ontology lead to relevant changes in other ontologies? This is a bit unclear to us.

**Response:** The work on the ontology will not directly lead to changes in other ontologies, as these are often led by different development groups. We indicate that we will submit changes for the consideration of other ontology developers in the following sentence in Step 2, but this sentence has been updated to be clearer:

- “ *Where we identify that *changes are needed to an external ontology *, we will log the **suggested** changes on the ontology’s GitHub repository **for the external developers’ consideration***.”

 
**Page 9**

***Step 3: Identifying and refining entities needed to structure the upper level of the ontology***

- In general, we believe it is very important for the authors to ensure that the upper-level entities sufficiently broad

**Response:** Thank you for the feedback. Our aim is to have entities that are broad enough to capture evidence about mental health across various contexts and studies.  

- Regarding presenting the first draft of the entities – Would it be feasible for the wider GALENOS team and advisory board to provide suggestions for upper-level entities before they are presented with the first draft. This to make sure that important aspects are not left out early in the process.

**Response:** We received feedback on the first draft of the upper level in a meeting with the advisory board and by sharing the upper level to the wider GALENOS team, which included asking them whether we missed any entities. The format allowed us to structure the discussion. However, in the future, a more open-ended format could be used to allow stakeholders to suggest initial entities. This limitation will be communicated in the future paper on developing the upper level of the GALENOS Mental Health Ontology. ***Step 4: Iterative stakeholder consultations of the Mental Health Ontology***

- Can the authors specify stakeholder criteria?

**Response:** We added this information on the criteria to Step 4 in the protocol:

- “ *The inclusion criteria will be having professional or volunteering experience on mental health project, being able to read and write in English and having access to an electronic device*.”

 

- How will the authors ensure stakeholders are well represented in terms of education level, age and gender etc?

**Response:** We do not have specific requirements regarding the representativeness regarding education level, age and gender. A key consideration during recruitment will be on encouraging people from underrepresented geographies to participate. We will contact the two lived experience advisors on our Advisory Board to forward the invitation to their networks for this. To specify this more clearly, the following addition has been made for the recruitment strategy:

- *Participants will be recruited by (1) inviting members of the Mental Health Ontology Advisory Board, (2) asking these members to suggest individuals or groups with relevant expertise, **with the two lived experience advisors being asked separately to share the invitation with their networks **…”*

 

- Regarding stakeholder survey – consider presenting response options in the protocol paper.

**Response:** Details on the response options in the stakeholder consultation have been added to the Methods Section Step 4:

- “ *Participants will be able to indicate which entities need changing by clicking options ‘Change label’ or ‘Change definition’ for the respective entities and providing open-ended feedback on how these should be changed. They will also be able to indicate that entities are missing or that relationships need changing in an open-ended response format. Finally, participants will be asked if they have any additional feedback which was not prompted by other survey questions.”*

**Pages 10-11**

- Step 8 (Evaluating the ontology): More information on the participants is required. Who are they? How many? Which groups will be represented?

**Response:** Details on the prospective participants has been added to Step 8 on the usability evaluation:

- “ *In line with stakeholder consultations on ontologies (3–29 participants) as part of the BCIO ( Michie et al., 2020; Norris et al., 2020) and relevant usability studies (Bruun & Stage, 2015), we will aim to recruit at least 10 participants. As the repository is likely to be used by people interested in data synthesis, the criteria will be for participants to have experience contributing to scoping or systematic reviews or applying review evidence in work related to mental health. To ensure that we include participants from a range of backgrounds, we will ask the lived experience advisors of the GALENOS Project to circulate invitations to their networks*.”

 

- Do members of the GALENOS team overlap with the author team? Please specify

**Response:** The ontology development team include Micaela Santilli, Janna Hastings, Susan Michie and Paulina Schenk, while Jennifer Potts is the project manager of the GALENOS Project, and Jaycee Kennett and Claire Friedrich are leads on systematic reviews. Initials have been added throughout to provide more clarity on the authors’ involvement in various steps.  

- It remains unclear how the researchers responsible for the development of the Mental Health Ontology´s will discuss the feedback and changes suggested by stakeholders (page 10, first paragraph under the heading “Analysis of stakeholder consultations”). This could be briefly clarified in one sentence with supporting references.

**Response:** The discussions will be informed by the specific feedback received; they will involve how the feedback will be addressed and why we have opted for this approach. The sentence has been updated to more clearly reflect this:

- “ *The relevant feedback and proposed changes will be discussed among the researchers leading the Mental Health Ontology’s development (JH, MS, SM & PS). **In these discussions, the team will consider how the feedback will be addressed and review disagreements between stakeholder comments, documenting the rationale for implementing relevant changes**.”*

**Page 11**

- “Applying the ontology to develop tools for data searching, visualising, extraction and synthesis, and partial automation of these processes the Mental Health Ontology will be used for annotations of the ‘living evidence’ extracted from the literature and stored in the project’s online data repository.” It is unclear to us what this section means - especially regarding the living evidence. Can the authors please rephrase or clarify?

**Response:** Thank you for noting this. We updated the title to reflect that we will not be able to develop automated extraction systems at this stage: “Applying the ontology to develop tools for data searching, visualising and synthesis, and partial automation of these processes” The first sentence has also been updated to be clearer:

- “ *The Mental Health Ontology will be used to organise the evidence extracted from the literature in the living systematic reviews and stored in the project’s online data repository (see Steps 2, 7 and 8).”*

We deleted the following sentence to reduce confusion and reflect the scope of the work more realistically:

- “ *In conjunction with machine learning algorithms and the data from living systematic reviews carried out early in the GALENOS Project’s lifecycle, the ontology will also be applied to develop and test structured search strategies for later systematic reviews.”*

  References Bruun, A., & Stage, J. (2015). An Empirical Study of the Effects of Three Think-Aloud Protocols on Identification of Usability Problems. In *Human-Computer Interaction – INTERACT 2015: 15th IFIP TC 13 International Conference* (pp. 159–176). https://doi.org/10.1007/978-3-319-22668-2_14 Brooke, J. (1996). SUS-A quick and dirty usability scale. *Usability evaluation in industry*, *189*(194), 4-7. Cipriani, A., Seedat, S., Milligan, L., Salanti, G., Macleod, M., Hastings, J., ... & Elliott, J. H. (2023). New living evidence resource of human and non-human studies for early intervention and research prioritisation in anxiety, depression and psychosis. *BMJ mental health*, *26*(1). Collaborators, G. M. D. (2022). Global, regional, and national burden of 12 mental disorders in 204 countries and territories, 1990-2019: a systematic analysis for the Global Burden of Disease Study 2019. *Lancet Psychiatry*, *9*(2), 137-50. Kennett, J., Friedrich, C., Chiocchia, V., Blackwell, S. E., Furukawa, T., Carlbring, P., ... & Cipriani, A. (2025). Cognitive bias modification for social anxiety: protocol for a living systematic review of human studies and meta-analysis. *Wellcome Open Research*, *9*, 657.

# Reviewer report: Reviewer response for version 2

**DOI:** 10.21956/wellcomeopenres.25788.r111650

**Published:** 2024-12-24

**Authors:** Carla Sharp

**Affiliations:** 1 University of Houston, Houston, USA

The authors have been sufficiently responsive to my comments.

Is the study design appropriate for the research question?

Partly

Is the rationale for, and objectives of, the study clearly described?

Partly

Are sufficient details of the methods provided to allow replication by others?

Partly

Are the datasets clearly presented in a useable and accessible format?

Not applicable

Reviewer Expertise:

Classification, assessment, treatment development.

I confirm that I have read this submission and believe that I have an appropriate level of expertise to confirm that it is of an acceptable scientific standard.

# Reviewer report: Reviewer response for version 1

**DOI:** 10.21956/wellcomeopenres.22909.r84245

**Published:** 2024-06-20

**Authors:** Carla Sharp

**Affiliations:** 1 University of Houston, Houston, USA

The goal of this paper is to outline the steps that the GALENOS project will engage in to develop a Mental Health Ontology. Drawing on Wright et al. (2022) which in turn, drew on several other indexed by this group (see any Michie articles on the development of the BCIO), the current paper outlines 6 steps that if completed will have resulted in the development of a Mental Health Ontology.

 In all, the goal of this work is much needed and of great potential significant. The paper is clearly written and the steps for ontology development broadly mirror best practices for ontology development. I think the paper can be improved by address what I perceive to be some gaps that may negatively affect  buy-in of this project - which we know is a significant prerequisite for ontology development and use (see recent NASEM report on the use of ontologies to accelerate behavioral sciences).

 1) It is not clear why the focus is on anxiety, depression and psychosis. Broadly speaking these three classes of disorders represent (however, not fully) an internalizing dimension of psychopathology, as well as psychoticism. Disorders representing the externalizing spectrum are not included; in addition, there is much debate about where personality disorders fit in into any of these dimensions, as well as somatoform disorders. Given high comorbidity between internalizing, externalizing, psychoticism, somatoform, personality, and trauma-related disorders, the applicability of a mental health ontology focused on depression, anxiety and psychosis is not clear to me. Other classes of disorders such as neurocognitive disorders are also not considered, calling into question the overall legitimacy of a mental health ontology that is very selective, thus neglecting to articulate relationships between specified (and missing) entities in the ontology.

 2) Related, the paper as it stands, seem to be unaware of the significant tensions between the clinical science the paper appears to rely on (HiTOP, or the personality-psychopathology spectrum approach) and other approaches to psychiatric nosology. If the authors are not aware of these tensions it may be important to become aware of them otherwise their selection of stakeholders and domain experts may be biased such that only a small portion of the clinical science community's views will be represented. Much of what Hi TOP proposes is interesting, but much work is still to be done before this framework becomes the sole guidance to the development of a mental health ontology.

 3) Related, I do not think 10 members provide enough options to fully represent views. It may be that the authors intend to make this a HiTOP-like ontology only, which is fine, and in which case 10 may be enough, but they will need to know that there may have to articulate commitment to this lens with the associated caveats that come with that.

 4) The timeline is unclear. To my mind, what the authors propose is highly ambitious, and it would be good to have a better sense of timeline, and the exact processes stakeholders and content experts will be engage in. Wright is reference, which in turn references the BCIO development, so this is a bit circular, and I'm wondering if other gold standard approaches to ontology development could strengthen (or expand) on the current proposed steps.

 5) A goal of the proposed mental health ontology is to explicate mechanisms. While I agree this is an important gap, I'm not sure what entities would be considered mechanisms given the broader literature (HiTOP) covered in the paper. I'm struggling to connect the dots with the type of descriptive psychiatry the authors espouse with a desire to also elucidate mechanisms.

 6) I was curious why the authors did not reference the recent NASEM report on the use of ontologies to accelerate behavioral sciences. The recommendations of the report, which single out BCIO as a strong example for the development of behavioral ontologies, would be in alignment with the proposed work.

Is the study design appropriate for the research question?

Partly

Is the rationale for, and objectives of, the study clearly described?

Partly

Are sufficient details of the methods provided to allow replication by others?

Partly

Are the datasets clearly presented in a useable and accessible format?

Not applicable

Reviewer Expertise:

Classification, assessment, treatment development.

I confirm that I have read this submission and believe that I have an appropriate level of expertise to state that I do not consider it to be of an acceptable scientific standard, for reasons outlined above.

# Response

**Published:** 2024-11-06

**Authors:** Paulina Margarete Schenk

**Affiliations:** University College London, London, England, UK

The goal of this paper is to outline the steps that the GALENOS project will engage in to develop a Mental Health Ontology. Drawing on Wright et al. (2022) which in turn, drew on several other indexed by this group (see any Michie articles on the development of the BCIO), the current paper outlines 6 steps that if completed will have resulted in the development of a Mental Health Ontology.

 In all, the goal of this work is much needed and of great potential significant. The paper is clearly written and the steps for ontology development broadly mirror best practices for ontology development. I think the paper can be improved by address what I perceive to be some gaps that may negatively affect  buy-in of this project - which we know is a significant prerequisite for ontology development and use (see recent NASEM report on the use of ontologies to accelerate behavioral sciences).

**Response:** We thank the reviewer for their feedback about this paper. All comments made by the reviewer have been addressed and implemented where appropriate.

 1) It is not clear why the focus is on anxiety, depression and psychosis. Broadly speaking these three classes of disorders represent (however, not fully) an internalizing dimension of psychopathology, as well as psychoticism. Disorders representing the externalizing spectrum are not included; in addition, there is much debate about where personality disorders fit in into any of these dimensions, as well as somatoform disorders. Given high comorbidity between internalizing, externalizing, psychoticism, somatoform, personality, and trauma-related disorders, the applicability of a mental health ontology focused on depression, anxiety and psychosis is not clear to me. Other classes of disorders such as neurocognitive disorders are also not considered, calling into question the overall legitimacy of a mental health ontology that is very selective, thus neglecting to articulate relationships between specified (and missing) entities in the ontology.

**Response:** The ontology’s scope and focus of development are shaped by that of the broader GALENOS Project. At this stage, we are focusing on developing an ontology that is sufficient for evidence synthesis in the GALENOS Project. We have made changes to this protocol’s Introduction to describe the GALENOS Project more clearly, as well as the role of the ontology within this project and how it will be applied. The reason that the ontology will initially focus on anxiety, depression and psychosis is due to the GALENOS Project’s focus on these areas. These three broad conditions were selected for the GALENOS Project as they have been identified as having the highest prevalence among mental health conditions and high mortality rates associated with them. We will also develop a broader upper-level structure so that we or others can develop other parts of the ontology relevant to different conditions over time (see the Methods section Step 3). We have reframed the title and content of the protocol to better reflect that we are working towards an ontology of mental health through addressing these specific focus areas first, rather than aiming to develop a fully comprehensive ontology at this stage. This project is now framed as the first step towards a more comprehensive ontology, which more realistically reflects the scope of the current work and presents the ontology’s development as primarily serving a particular use case at this stage. As the reviewer points out, anxiety, depression and psychosis are very broad conditions with some disagreements as to what “disorders” fall underneath them. Our intention is to remain agnostic regarding any specific classification system, to represent conditions as diagnoses rather than disorders to remain compatible with different diagnostic systems, and to explicitly indicate symptoms where we can. Thereby, the ontology will remain open to the possibility that diagnoses of mental health conditions may be assigned based on different classification frameworks in different contexts. Entities will be developed as broadly as is needed to fulfil the objective of capturing, comparing and integrating evidence based on the research questions of the systematic reviews in the GALENOS Project. Feedback from a broader group of stakeholders will be used to further develop and refine entities.

 2) Related, the paper as it stands, seem to be unaware of the significant tensions between the clinical science the paper appears to rely on (HiTOP, or the personality-psychopathology spectrum approach) and other approaches to psychiatric nosology. If the authors are not aware of these tensions it may be important to become aware of them otherwise their selection of stakeholders and domain experts may be biased such that only a small portion of the clinical science community's views will be represented. Much of what Hi TOP proposes is interesting, but much work is still to be done before this framework becomes the sole guidance to the development of a mental health ontology.

**Response:** We appreciate the tensions in clinical science between traditional diagnostic approaches and the dimensional approaches that focus on experiences within context (e.g., as proposed by HiTOP, or the symptom network approaches). We understand that there remains work to be done to develop these alternative approaches. Our objective is to provide a framework that can be used in an integrative fashion. The reason for explicitly noting classification systems and frameworks with differing perspectives was to represent entities that can be used to integrate evidence from a wide range of different sources. However, we appreciate the need for the project scope to be more realistic within the specified timeframe. We have removed the mapping work to classification systems (specified in Step 3) from the workflow and protocol. In the Conclusion section, we have noted that future work can involve mapping the ontology to existing classification systems to improve cross-referencing to these systems and to ensure that entities relating to mental health are comprehensively captured.

 3) Related, I do not think 10 members provide enough options to fully represent views. It may be that the authors intend to make this a HiTOP-like ontology only, which is fine, and in which case 10 may be enough, but they will need to know that there may have to articulate commitment to this lens with the associated caveats that come with that.

**Response:** We have currently 17 advisory board members from 10 different countries and have been inviting additional people to join when additional expertise or perspectives are identified. Members include people with expertise from various domains, including psychiatry, clinical psychology and health psychology, as well as lived experience. Additions have been made to the section “ *Set up the Mental Health Ontology Advisory Board*” to provide an overview of current advisory board members. The stakeholder consultations will allow us to receive additional feedback from a wider range of people. For the stakeholder reviews, we are aiming to have feedback from at least 10 participants, as the tasks can be long and require considerable expertise in some cases (e.g., a good grasp of both the mental health domain and ontologies). This can make recruiting more participants quite challenging. We appreciate that feedback from more people would help ensure that the ontology is more representative of wider views in mental health. In addition to the stakeholder consultations and advisory board, ontologies are meant to be updated based on feedback over time. Updates have been made to the Methods section Step 4 to more clearly specify the stakeholder consultations. Finally, the GitHub repository, where we will maintain the ontology, allows users to report issues with the ontology. This functionality allows us to track issues with the ontology over time and ensure that the ontology is updated in line with wider perspectives. An additional project on developing related ontologies has been funded for 2024-2029 and we will be tracking any issues with the Mental Health Ontology throughout this period and making updates accordingly. Updates have been made to the Conclusion sections of the protocol to explain the plans for the ontology’s maintenance.

 4) The timeline is unclear. To my mind, what the authors propose is highly ambitious, and it would be good to have a better sense of timeline, and the exact processes stakeholders and content experts will be engage in. Wright is reference, which in turn references the BCIO development, so this is a bit circular, and I'm wondering if other gold standard approaches to ontology development could strengthen (or expand) on the current proposed steps. **Response:** The timeline of the project, three years, will be added to the protocol (see Methods section Step 2). As we appreciate that the proposal was highly ambitious, we have updated the workflow to be more realistic within the given timeline. For example, the work to map the ontology to mental health classification systems has been removed from our Methods, as this work was not essential for the ontology’s use case in the GALENOS Project. As the reviewer suggests, stakeholder consultations every 6-9 months was too ambitious given the resources available, and therefore, we have adjusted our timelines. For each set of three systematic reviews conducted annually, stakeholders will be asked to provide feedback on the clarity of entities mapped onto these reviews, as these will be used to structure the GALENOS data repository. However, stakeholders will only be asked to review entities that have not been included in a previous consultation. In addition, we will have one stakeholder consultation on the upper level of the ontology. Updates have been made to the Methods section Step 4 in the protocol to reflect this change. A key difference of this work to the BCIO development process is that we are working on an ontology to be fit for a specific use case in the GALENOS Project. The overview of the Methods section will be updated to clarify this difference, and emphasise the application of the Mental Health Ontology in the context of the GALENOS Project.  

 5) A goal of the proposed mental health ontology is to explicate mechanisms. While I agree this is an important gap, I'm not sure what entities would be considered mechanisms given the broader literature (HiTOP) covered in the paper. I'm struggling to connect the dots with the type of descriptive psychiatry the authors espouse with a desire to also elucidate mechanisms.

**Response:** We will draw on diverse perspectives about mental health, without trying to position the ontology as linked to a particular perspective. The protocol has been updated to avoid any confusion resulting from references to HiTOP. In the upper-level structure of the ontology, we will include an entity for “mental health intervention mechanisms of action” to conceptually structure the ontology and allow users to describe or synthesise evidence about mechanisms. We conceptualise mechanisms as processes that bring about the effect of an intervention on an outcome (e.g., beliefs, opportunities, bodily processes). As intervention outcomes can be very different in mental health interventions, such mechanisms can be extremely diverse as well. We will prioritise developing entities for mechanisms of action that are identified as part of the living systematic reviews in the GALENOS Project, but construct these in such a way that they can be extended to other conditions over time.

 6) I was curious why the authors did not reference the recent NASEM report on the use of ontologies to accelerate behavioral sciences. The recommendations of the report, which single out BCIO as a strong example for the development of behavioral ontologies, would be in alignment with the proposed work.

**Response:** As the reviewer suggests, the reference was added to the Introduction, as it is highly relevant to the current work.   We thank the reviewer for their useful suggestions, and the questions they raised.

# Reviewer report: Reviewer response for version 1

**DOI:** 10.21956/wellcomeopenres.22909.r79166

**Published:** 2024-05-06

**Authors:** Maya Braun

**Affiliations:** 1 Department of Experimental-Clinical and Health Psychology, Ghent University, Gent, Belgium

The GALENOS project aims to develop a Mental Health Ontology to facilitate knowledge aggregation and evidence synthesis. Their methodology for ontology is based on existing classification frameworks and stakeholder consultation.

 The paper is a highly relevant roadmap for researchers interested in using ontologies within the behavioural sciences. It provides an outlook on the GALENOS project and the development of the mental health ontology. As a protocol for ontology development, some information could be provided in more detail.

**Methodology**

 The project uses a methodology based on existing classification systems, systematic reviews and stakeholder involvement, similar to processes in existing ontologies created in the context of the Human Behaviour Change Project. The methodology seems largely suited for this project, though some aspects remain unclear.

 First and most importantly, is not clear to me how the finalised ontology will be evaluated and what the maintenance plan is for the ontology.

 Second, it is unclear to me which GALENOS teams there are, and what the background of the researchers in the different teams is. Please provide more information on this, or link to further information. Similarly, it is not clear to me how many systematic reviews there are, and what they focus on.

 Third, preliminary information regarding the external ontologies you are planning to link to would be valuable, particularly since you incorporate collaboration with the ontology developers. Working together closely with developers of external ontologies might not always be possible. How will reuse of entities be handled if close collaboration is not possible?

**Background**

 In general, the background of the project is clearly described, albeit short. I believe the background would benefit from some revisions to further clarify the goal of the project.

 First, the choice of anxiety, depression and psychosis specifically is not sufficiently justified in the background text. Relatedly, the focus shift from “anxiety, depression and psychosis” to “a broad range of aspects of mental health” is unclear to me. Do I understand correctly that the ontology covers all mental health related diagnoses, but the reviews focus on anxiety, depression and psychosis? It is also unclear to me how the mental health ontology relates to non-human animals. Please clarify this in the text.

 Second, the introduction uses a lot of technical terms that readers with a behavioural sciences background might not be familiar with. Vocabulary regarding ontologies are not sufficiently clarified. Please provide some further information on what ontologies are and why they should be used in this context. It is also unclear to me how the cross-references between different diagnostic systems will work, and how this will improve upon current classification systems. Please also provide additional information regarding the planned “reliable machine learning” – does this refer to the language models mentioned on page 9?

Is the study design appropriate for the research question?

Partly

Is the rationale for, and objectives of, the study clearly described?

Partly

Are sufficient details of the methods provided to allow replication by others?

Partly

Are the datasets clearly presented in a useable and accessible format?

Not applicable

Reviewer Expertise:

Using ontologies in behaviour change (physical activity) interventions

I confirm that I have read this submission and believe that I have an appropriate level of expertise to confirm that it is of an acceptable scientific standard, however I have significant reservations, as outlined above.

# Response

**Published:** 2024-11-06

**Authors:** Paulina Margarete Schenk

**Affiliations:** University College London, London, England, UK

The GALENOS project aims to develop a Mental Health Ontology to facilitate knowledge aggregation and evidence synthesis. Their methodology for ontology is based on existing classification frameworks and stakeholder consultation.

 The paper is a highly relevant roadmap for researchers interested in using ontologies within the behavioural sciences. It provides an outlook on the GALENOS project and the development of the mental health ontology. As a protocol for ontology development, some information could be provided in more detail.

**Response:** We thank the reviewer for their feedback about this paper. All comments made by the reviewer have been addressed and implemented where appropriate.

**Methodology**

 The project uses a methodology based on existing classification systems, systematic reviews and stakeholder involvement, similar to processes in existing ontologies created in the context of the Human Behaviour Change Project. The methodology seems largely suited for this project, though some aspects remain unclear.

 First and most importantly, is not clear to me how the finalised ontology will be evaluated and what the maintenance plan is for the ontology.

**Response:** The Mental Health Ontology is being developed for a particular application to help integrate the data from the different living systematic reviews of the GALENOS Project in the associated data repository. The method described in the protocol has been updated to better reflect the application focus of the ontology. We will evaluate the usability of the ontology-structured data repository; information about this has now been added to the protocol as Step 8 in the Methods section. Furthermore, the ontology will be evaluated through stakeholder reviews as described in the protocol. The Mental Health Ontology will be maintained alongside the wider Behaviour Change Intervention Ontology as part of a 5-year NIH grant on increasing the usability of ontologies. The ontology will be open to feedback on GitHub, and the research team associated with the grant will monitor the comments to make appropriate updates. Information about this has been added to the Conclusion section of the protocol. The ontology’s application as part of the data repository will be maintained by the relevant team, Future Evidence, of the GALENOS Project.

 Second, it is unclear to me which GALENOS teams there are, and what the background of the researchers in the different teams is. Please provide more information on this, or link to further information. Similarly, it is not clear to me how many systematic reviews there are, and what they focus on.

**Response:** The goal of the GALENOS project is to carry out systematic reviews in sets of around three per year for human studies and three per year for animal studies until the completion of the project (planned as January 2026). However, the exact number of the systematic reviews has not been specified. This information has been added to the Methods section Step 2 in the current protocol. The researchers are based in countries spanning several continents, with backgrounds in psychiatry, psychology, data science and expertise relating to lived experience. The reviews are led by researchers with an MSc or PhD relating to psychology or psychiatry. Each stage of the review received input from the leadership team and Global Lived Experience Advisory Board. The leadership team includes members from Australia, several European countries, Japan and South Africa. Global Lived Experience Advisory Board includes members from Canada, India, Nigeria, the Philippines and Zimbabwe. The project members are clinicians, experts by lived experience, and researchers with expertise across data science, psychology and psychiatry. Detailed information about the GALENOS Project can be found on the project website ( https://www.galenos.org.uk/about), as well as in the protocol for the overarching project ( https://mentalhealth.bmj.com/content/ebmental/26/1/e300759.full.pdf). This information has been added to relevant parts of the Introduction and Methods section Step 2 in the current protocol.

 Third, preliminary information regarding the external ontologies you are planning to link to would be valuable, particularly since you incorporate collaboration with the ontology developers. Working together closely with developers of external ontologies might not always be possible. How will reuse of entities be handled if close collaboration is not possible? **Response:** We are reusing relevant parts of the Behaviour Change Intervention Ontology (BCIO), the Mental Functioning Ontology (MFO), Emotion Ontology (MFEOM), Information Artifact Ontology (IAO) and where necessary, a small number of classes from other ontologies available on the EMBL-EBI Ontology Lookup Service ( https://www.ebi.ac.uk/ols4/). Information about this has been added to the Methods section Step 2. As the reviewer points out, close collaboration might be difficult with external ontology developers, both due to their time constraints and the current project’s timelines. We will submit feedback on the appropriate GitHub issue trackers for the developers of other ontologies to address, in cases where we are reusing entities, but these entities need some updates. In some cases, we may decide not to reuse entities (e.g., if they do not capture the intended meaning of a construct in a review or do not fit into the ontology’s structure), instead developing more fitting entities. Where relevant (e.g., entities closely overlap with entities in external ontology), we will inform the external ontology developers via GitHub. Details about this approach have been added to the Method section Step 2. Any mentions of ‘collaboration’ have been removed where close collaboration is not possible, in order to avoid confusion. In cases where the external ontologies are co-developed by members of the project team (BCIO, Mental Functioning and Emotion Ontologies), we expect good collaboration to be possible. In addition, we have good experiences of collaboration with the developers of ontologies that are actively maintained and participate in communities such as the Open Biological and Biomedical Ontology (OBO) Foundry.  

**Background**

 In general, the background of the project is clearly described, albeit short. I believe the background would benefit from some revisions to further clarify the goal of the project.

 First, the choice of anxiety, depression and psychosis specifically is not sufficiently justified in the background text.

**Response:** Additions have been made to more clearly describe the GALENOS Project in the Introduction section. The reason for focusing on anxiety, depression and psychosis within the GALENOS Project is the high prevalence and impact of these mental conditions globally. As this is the focus of the broader project as a whole, the Mental Health Ontology will also focus on these three conditions, but the upper-level structure of the ontology will be developed in a way that is compatible with other conditions as well. The title and framing of the protocol have been updated to better reflect that the work involves a step towards an ontology of mental health in the context of the GALENOS project’s focus areas (anxiety, depression and psychosis), rather than developing a comprehensive ontology of mental health. This reframing will better show the scope of the current work and help illustrate the development of the ontology mostly within the context of a particular use-case, structuring and integrating evidence regarding anxiety, depression and psychosis.  

 Relatedly, the focus shift from “anxiety, depression and psychosis” to “a broad range of aspects of mental health” is unclear to me. Do I understand correctly that the ontology covers all mental health related diagnoses, but the reviews focus on anxiety, depression and psychosis?

**Response:** As noted above, the Mental Health Ontology is being developed so that the upper-level structure (e.g., upper levels, such as human population, mental health intervention) can be used for all mental health conditions. As the reviewer correctly noted, the framing of the protocol needs to be updated to better reflect that this ontology will be developed within a use-case. The GALENOS Project’s systematic reviews focus on anxiety, depression and psychosis. Accordingly, the content of the ontology will also primarily relate to these conditions and the research questions that are associated with the reviews. We have clarified in the Introduction that ontologies are always in development, and that what we are doing is creating an overall structure for representing knowledge about mental health but elaborating in detail three areas relevant to mental health: anxiety, depression and psychosis.  

 It is also unclear to me how the mental health ontology relates to non-human animals. Please clarify this in the text.

**Response:** The GALENOS Project is piloting an innovative approach in which each living systematic review includes relevant evidence based on human studies as well as evidence based on animal studies. Accordingly, both human and non-human content will be included in the online data repository and may be annotated to the ontology terms where possible. However, as animal-specific research is already included in the scope of other ontologies in the biomedical domain, animal-specific entities will not be explicitly included in the Mental Health Ontology. To avoid confusion, this part of the protocol has been omitted.    

 Second, the introduction uses a lot of technical terms that readers with a behavioural sciences background might not be familiar with. Vocabulary regarding ontologies are not sufficiently clarified. Please provide some further information on what ontologies are and why they should be used in this context.

**Response:** More details have been added to the Introduction to clarify what ontologies are and how the current ontology will be useful in context of the GALENOS Project. A figure (see newly added Figure 1) illustrating the link between the ontology development and application within the context of the GALENOS Project has also been added.  

 It is also unclear to me how the cross-references between different diagnostic systems will work, and how this will improve upon current classification systems.

**Response:** Categories from different diagnostic systems can be mapped onto the same entity in the ontology where relevant, and these categories can be signposted using an appropriate “cross-reference” annotation for an entity. The ontology does not intend to replace existing classifications systems. Instead, the ontology will enable evidence that has been gathered in multiple of those classification systems to be integrated and inter-related. The ontology will propose an unambiguous definition for each entity, with concepts from different systems integrated into the ontology. We have removed Step 3 (Identifying and refining entities and their structure based on existing classification systems) from the protocol, in response to other comments on the scope and timeline of the work. For the current phase of the project, our focus is on the entities that are required for the data repository. In the Conclusion, we have noted that further work will be required to comprehensively map the ontology onto existing classification systems to improve cross-referencing and ensure that entities relating to mental health are well captured in the ontology.  

 Please also provide additional information regarding the planned “reliable machine learning” – does this refer to the language models mentioned on page 9?

**Response:** The phrase “reliable machine learning” is used to describe machine learning algorithms that have been tested and found to produce reliable results. To reduce confusion, the phrase “reliable machine learning” has been amended to “machine learning algorithms”. In addition, as the reviewer points out, the phrase “language models” on page 9 is used to describe one of the machine learning approaches that can be used with ontologies. For consistency, we have updated the phrase “language models” to the more general phrase “machine learning algorithms” on page 9. The computer science team will test out different machine learning approaches depending on the needs of the project. A clarification has been added to the protocol that this will be an iterative process, depending on the evolving deliverables of the GALENOS Project (see section “ *Applying the ontology to develop tools for data searching, visualising, extraction and synthesis, and partial automation of these processes*”).   We thank the reviewer for their useful suggestions, and the questions they raised.
